# Supplementary material for: The evolution of centriole degradation in mouse sperm
Source: Nat Commun. 2024 Jan 2;15:117. doi: 10.1038/s41467-023-44411-8 (PMC10761967; doi:10.1038/s41467-023-44411-8)
Supplement: Supplementary file 1 — Supplementary Information file [file 41467_2023_44411_MOESM1_ESM.pdf]

Supplementary figure with grayscale single panels, Supplementary Figures, Supplementary tables, and their Legends

Supplementary Figures 5, 6, and 7 with grayscale single panels

Fig 5

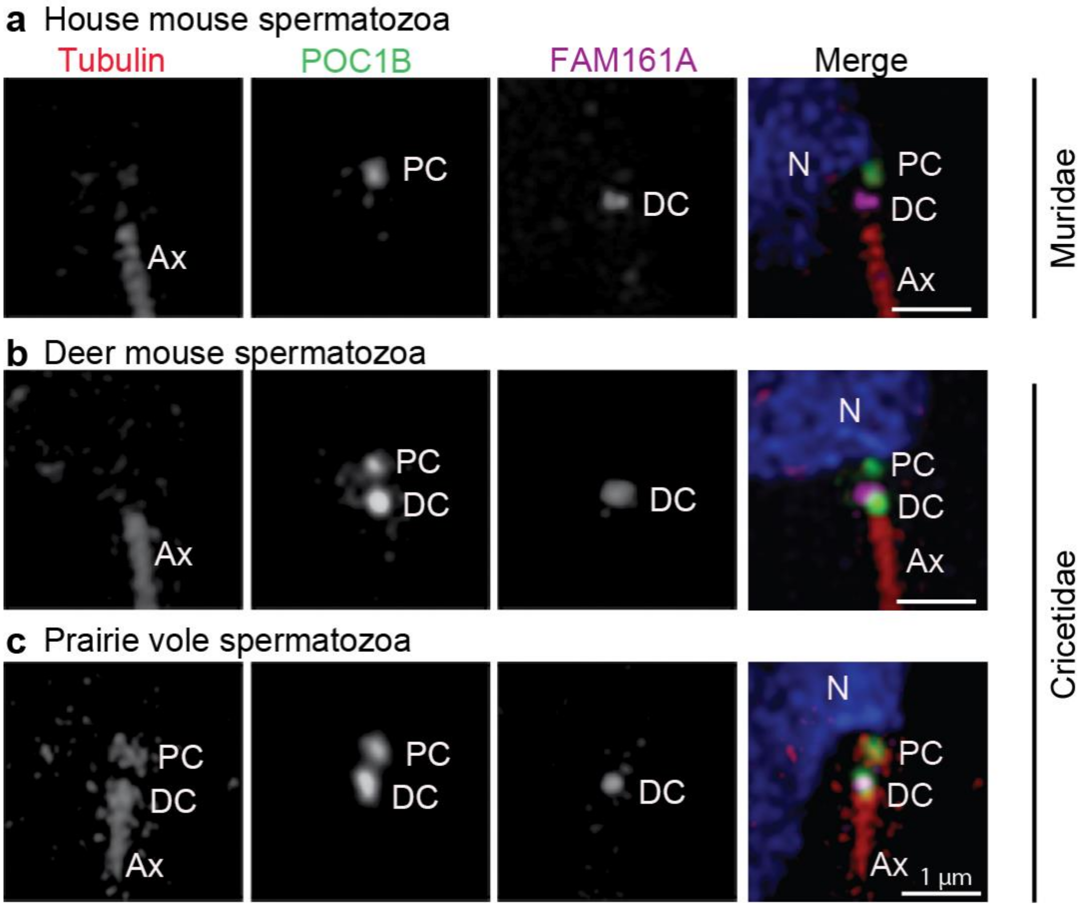

Fig 6

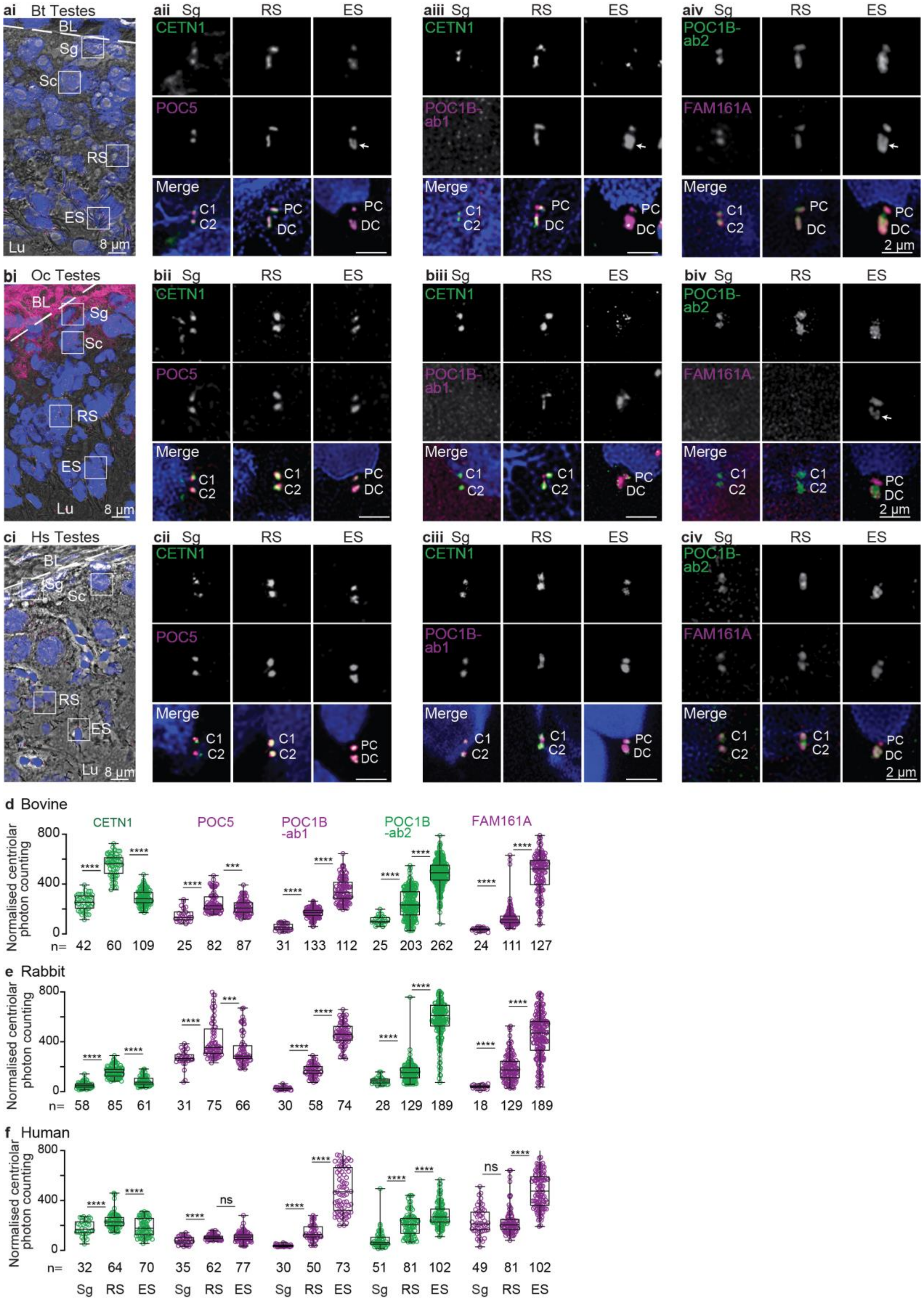
ai Bt Testes
BL
Sg
Sc
RS
ES
Lu
8 µm
aii Sg RS ES
CETN1
POC5
Merge
C1
C2
PC
DC
aiii Sg RS ES
CETN1
POC1B-ab1
Merge
aiv Sg RS ES
POC1B-ab2
FAM161A
Merge
2 µm
bi Oc Testes
bii Sg RS ES
biii Sg RS ES
biv Sg RS ES
ci Hs Testes
cii Sg RS ES
ciii Sg RS ES
civ Sg RS ES
d Bovine
CETN1
POC5
POC1B -ab1
POC1B -ab2
FAM161A
Normalised centriolar photon counting
800
400
0
n= 42 60 109 25 82 87 31 133 112 25 203 262 24 111 127
e Rabbit
n= 58 85 61 31 75 66 30 58 74 28 129 189 18 129 189
f Human
ns
n= 32 64 70 35 62 77 30 50 73 51 81 102 49 81 102
Sg RS ES Sg RS ES Sg RS ES Sg RS ES Sg RS ES

Fig 7

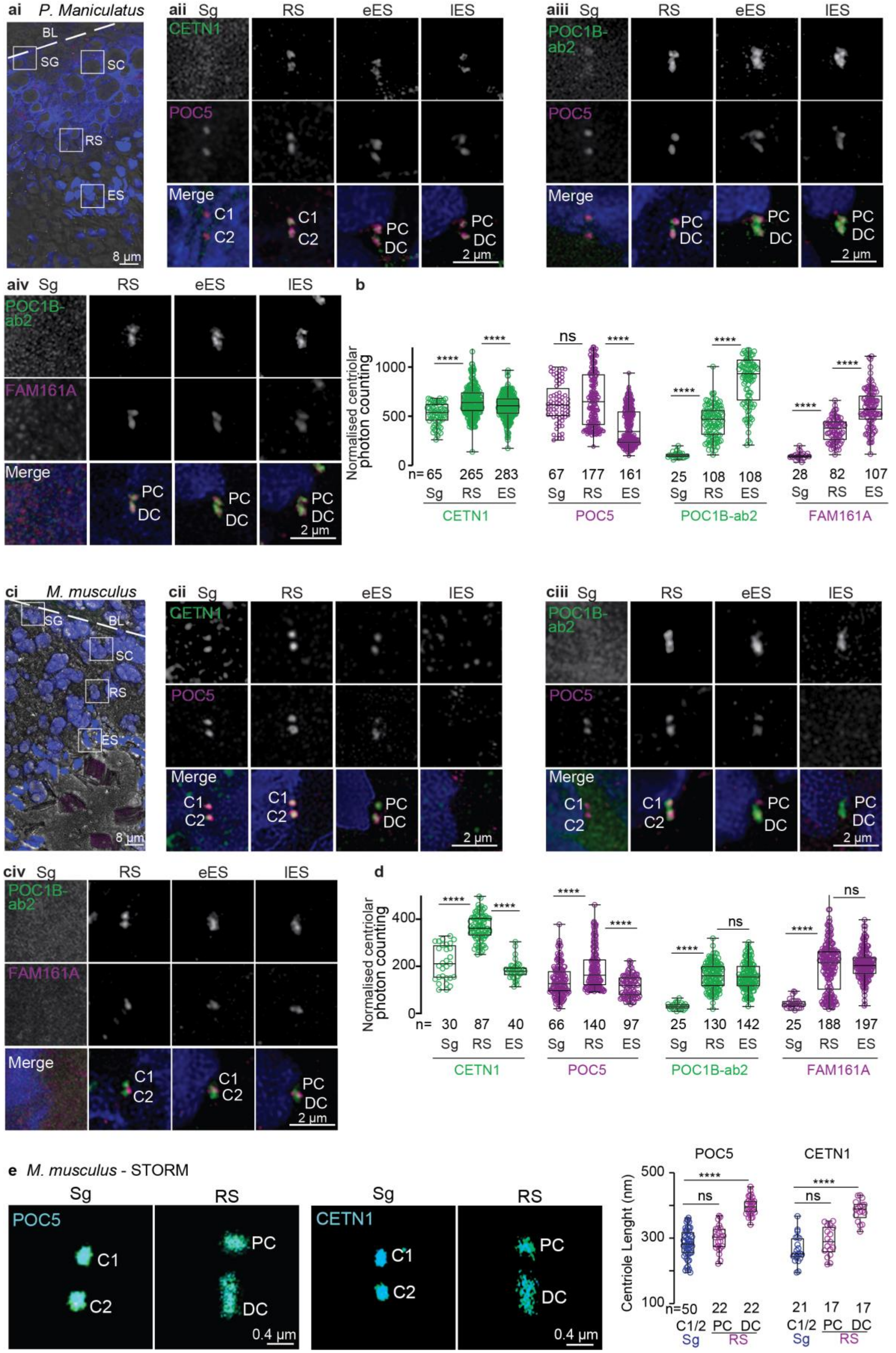
ai P. Maniculatus
BL
SG
SC
RS
ES
8 µm
aii Sg RS eES lES
CETN1
POC5
Merge
C1
C2
PC
DC
2 µm
aiii Sg RS eES lES
POC1B-ab2
POC5
Merge
PC
DC
2 µm
aiv Sg RS eES lES
POC1B-ab2
FAM161A
Merge
PC
DC
2 µm
b
Normalised centriolar photon counting
1000
500
0
n= 65 265 283 67 177 161 25 108 108 28 82 107
Sg RS ES Sg RS ES Sg RS ES Sg RS ES
CETN1
POC5
POC1B-ab2
FAM161A
****
ns
ci M. musculus
SG
BL
SC
RS
ES
8 µm
cii Sg RS eES lES
CETN1
POC5
Merge
C1
C2
PC
DC
2 µm
ciii Sg RS eES lES
POC1B-ab2
POC5
Merge
C1
C2
PC
DC
2 µm
civ Sg RS eES lES
POC1B-ab2
FAM161A
Merge
C1
C2
PC
DC
2 µm
d
Normalised centriolar photon counting
400
200
0
n= 30 87 40 66 140 97 25 130 142 25 188 197
Sg RS ES Sg RS ES Sg RS ES Sg RS ES
CETN1
POC5
POC1B-ab2
FAM161A
****
ns
e M. musculus - STORM
Sg RS Sg RS
POC5
C1
C2
PC
DC
0.4 µm
CETN1
C1
C2
PC
DC
0.4 µm
POC5
CETN1
Centriole Lenght (nm)
500
300
100
****
ns
n=50 22 22 21 17 17
C1/2 PC DC C1/2 PC DC
Sg RS Sg RS

Supplementary Figures, Supplementary Tables, and their Legends

Supplementary Figure 1– Spermatozoa with one canonical centriole (the proximal centriole) and one atypical centriole (the distal centriole) are present throughout the mammalian tree of life.

a) Example of paddle/eyedrop-shaped sperm head with a centrally inserted neck, barrel-shaped proximal centriole (PC, canonical centriole), and funnel-shaped distal centriole (DC, atypical centriole), as in humans, and a sickle-shaped sperm head with a laterally attached neck and no centrioles, as in house mice. Based on Knobil and Neill's Physiology of Reproduction [1]. The acrosome is shown in red; the nucleus is shown in blue, and all other parts of the sperm are shown in green. **b**) A systematic survey of previous transmission electron microscopic studies of Eutherian spermatozoan centrioles.

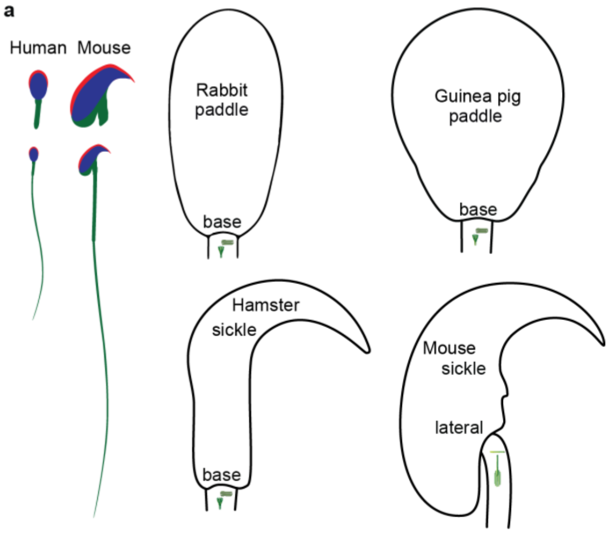

b **bi) Eutherian**: The four main groups of Eutherian mammalian species (color-coded) have one canonical centriole (the PC) in their spermatozoa. Phylogeny based on (Song et al., 2012).

Phylogeny: Boreoeutheria; Euarchontoglires (Superorder) - Order: Primates
- African green monkey (*Cercopithecus aethiops*) (Bedford, 1967)
- *Homo sapiens* (Pedersen, 1969) (Garanina et al., 2019)

Phylogeny: Boreoeutheria; Euarchontoglires (Superorder); Glires Order: Rodentia
- Siberian chipmunk (*Tamias sibiricus*) (Lee and Park, 2011)
- Chinchilla (Healey and Weir, 1970)
- Guinea Pig (Caviidae) (Fawcett, 1965)

Phylogeny: Boreoeutheria; Laurasiatheria (Superorder) - Order: Carnivora
- Cat (Schmehl and Graham, 1989) (Sato and Oura, 1984)

Phylogeny: Boreoeutheria; Laurasiatheria (Superorder) - Order: Chiroptera
- Bat (Bernard and Hodgson, 1988)

Phylogeny: Boreoeutheria; Laurasiatheria (Superorder) - Order: Eulipotyphla
- Lesser white-toothed shrew (*Crocidura suaveolens*) (Soon-Jeong et al., 2006)

Phylogeny: Boreoeutheria; Laurasiatheria (Superorder) - Order: Artiodactyla
- Boar (Nicander and Bane, 1962)
- Bovine (Ounjai et al., 2012)
- Camel ` (Tingari, 1991)

Phylogeny: Boreoeutheria; Laurasiatheria (Superorder) - Order: Perissodactyla
- Horse (Leung et al., 2021)

Phylogeny: Afrotheria; Order: Proboscidea
- Asiatic elephant (*Elephas maximus*) (Heath et al., 1983)

Phylogeny: Afrotheria Order: Macroscelidea
- Macroscelidea (elephant shrews) (Woodall and FitzGibbon, 1995)

Phylogeny: Xenarthra
- Six-banded armadillo (*Euphractus sexcinctus*) (Sousa et al., 2013)

bii) Monotreme: The structure of the sperm neck is simpler in Monotreme Mammals than in Eutherian Mammals: While a PC is found near the nucleus, and the mitochondria reach the nucleus, neither striated columns nor a canonical DC are observed (Carrick and Hughes, 1982).

biii) Marsupial: In Marsupials, the DC is not observed or persists in a modified form (Lloyd et al., 2002; Temple-Smith, 1994). The PC (aka transverse centriole) is observed in some species (Lloyd et al., 2002), having been reported in the mature spermatozoa of *Perameles nasuta*, *Macrotis lagotis*, several dasyurids, the petaurid genus Petaurus, and the peramelid *Isoodon macrourus* ((Sapsford et al., 1969) and (Johnston et al., 1995) in (Lloyd et al., 2002)). A remnant PC is present in *Hypsiprymnodon moschatus* (Lloyd et al., 2002). No PC is detected in *Trichosurus vulpecula* and most macropods ((Harding, 1979) in (Lloyd et al., 2002)).

b References:

Bedford, J.M. 1967. Observations on the fine structure of spermatozoa of the bush baby (Galago senegalensis), the African green monkey (Cercopithecus aethiops) and man. *The American journal of anatomy*. 121:443-459.

Bernard, R.T., and A.N. Hodgson. 1988. Fine structure of the neck of epididymal spermatozoa of Schreiber's long-fingered bat (Chiroptera: Mammalia). *Gamete Res*. 21:41-50.

Carrick, F.N., and R.L. Hughes. 1982. Aspects of the Structure and Development of Monotreme Spermatozoa and Their Relevance to the Evolution of Mammalian Sperm Morphology. *Cell and Tissue Research*. 222:127-141.

Fawcett, D.W. 1965. The anatomy of the mammalian spermatozoon with particular reference to the guinea pig. *Z Zellforsch Mikrosk Anat*. 67:279-296.

Garanina, A.S., I.B. Alieva, E.E. Bragina, E. Blanchard, B. Arbeille, F. Guerif, S. Uzbekova, and R.E. Uzbekov. 2019. The Centriolar Adjunct(-)Appearance and Disassembly in Spermiogenesis and the Potential Impact on Fertility. *Cells*. 8:180.

Harding, H.R. 1979. Reproduction in male marsupials. A critique with additional observation on sperm development and structure, Elibrary..

Healey, P., and B.J. Weir. 1970. Changes in the ultrastructure of chinchilla spermatozoa in different diluents. *J Reprod Fertil*. 21:191-193.

Heath, E., R. Jeyendran, and E. Graham. 1983. Ultrastructure of Spermatozoa of the Asiatic Elephnat (Elephas maximus). *Anatomia, Histologia, Embryologia*. 12:245-252.

Johnston, S., L. Daddow, and F. Carrick. 1995. Ultrastructural and light microscopic observations of mature epididymal spermatozoa and sperm maturation of the greater bilby Macrotis lagotis (Metatheria, Mammalia). *Advances in Spermatozoal Phylogeny and Taxonomy*. 166:397-407.

Lee, J.-H., and K.-R. Park. 2011. Fine Structure of Sperm in the Korea Squirrel, Tamias sibiricus. *Applied Microscopy*. 41:99-107.

Leung, M.R., M.C. Roelofs, R.T. Ravi, P. Maitan, H. Henning, M. Zhang, E.G. Bromfield, S.C. Howes, B.M. Gadella, H. Bloomfield-Gadelha, and T. Zeev-Ben-Mordehai. 2021. The multi-scale architecture of mammalian sperm flagella and implications for ciliary motility. *EMBO J*. 40:e107410.

Lloyd, S., F. Carrick, and L. Hall. 2002. Ultrastructure of the mature spermatozoon of the musky rat-kangaroo, Hypsiprymnodon moschatus (Potoroidae: Marsupialia). *Acta Zoologica*. 83:167-174.

Nicander, L., and A. Bane. 1962. Fine structure of boar spermatozoa. *Z Zellforsch Mikrosk Anat*. 57:390-405.

Ounjai, P., K.D. Kim, P.V. Lishko, and K.H. Downing. 2012. Three-dimensional structure of the bovine sperm connecting piece revealed by electron cryotomography. *Biology of reproduction*. 87:73.

Pedersen, H. 1969. Ultrastructure of the ejaculated human sperm. *Z Zellforsch Mikrosk Anat*. 94:542-554.

Sapsford, C., C.A. Rae, and K. Cleland. 1969. Ultrastructual studies on maturing spermatids and on sertoli cells in the bandicoot Perameles nasuts Geoffroy (Marsupialia). *Australian Journal of Zoology*. 17:195-292.

Sato, N., and C. Oura. 1984. The fine structure of the neck region of cat spermatozoa. *Okajimas folia anatomica Japonica*. 61:267-285.

Schmehl, M., and E. Graham. 1989. Ultrastructure of the domestic tom cat (Felisdomestica) and tiger (Pantheratigrisaltaica) spermatozoa. *Theriogenology*. 31:861-874.

Song, S., L. Liu, S.V. Edwards, and S.Y. Wu. 2012. Resolving conflict in eutherian mammal phylogeny using phylogenomics and the multispecies coalescent model. *Proceedings of the National Academy of Sciences of the United States of America*. 109:14942-14947.

Soon-Jeong, J., P. Joo-Cheol, K. Heung-Joong, S.B. Chun, Y. Myung-Hee, L. Do-Seon, and J. Moon-Jin. 2006. Comparative fine structure of the epididymal spermatozoa from three Korean shrews with considerations on their phylogenetic relationships. *Biocell*. 30:279-286.

Sousa, P.C., E.A. Santos, J.A. Bezerra, G.L. Lima, T.S. Castelo, J.D. Fontenele-Neto, and A.R. Silva. 2013. Morphology, morphometry and ultrastructure of captive six-banded armadillo (Euphractus sexcinctus) sperm. *Animal reproduction science*. 140:279-285.

Temple-Smith, P.D. 1994. Comparative structure and function of marsupial spermatozoa. *Reprod Fertil Dev*. 6:421-435.

Tingari, M. 1991. Studies on camel semen. III. Ultrastructure of the spermatozoon. *Animal reproduction science*. 26:333-344.

Woodall, P.F., and C. FitzGibbon. 1995. Ultrastructure of Spermatozoa of the Yellow-rumped Elephant Shrew Rhynchocyon chrysopygus (Mammalia: Macroscelidea) and the Phylogeny of Elephant Shrews. *Acta Zoologica*. 76:19-23.

Supplementary Table 1 – In rodents, FAM161A is evolving more quickly than the other rod proteins in Muridae.

**Gene**	*FAM161A* (Muridae)	*FAM161A* (Cricetidae)	*FAM161A* (Other Myomorpha)	*FAM161A* (Rodents)	*FAM161B* (Rodents)	*POC1b* (Rodents)	*POC5* (Rodents)	*CETN1* (Rodents)	*WDR90* (Rodents)
ω (dN/dS)	0.62960	0.57030	0.48616	0.57592	0.33602	0.19055	0.26702	0.03423	0.26510

Supplementary Table 2– Codon-wide selection analysis to identify sites under positive and negative selection.

Selection analysis identified more positively (Green font) and negatively (red font) selected sites in rodents than in primates, Carnivora, and ungulates. The number of codons used in each lineage is indicated in parentheses**.** The number of selected sites and the site numbers along the primary structure are indicated for each method and mammalian order. MEME [2] is a generalization of FEL [3]. The initial analytical phases of the two approaches are identical; however, FEL assumes that the same dN/dS (ω) ratio applies to all branches, while MEME models variable dN/dS (ω) values across lineages at an individual site.

**Selection Analysis**	**Ungulates** 722 codons	**Carnivora**722 c odons	**Primates** 716 codons	**Rodents** 714 codons
**MEME:** Episodic Diversifying Selection (P< 0.05)	**9 Sites**: 46, 47, 49, 60, 62, 67, 84, 312, 440, 505	**7 sites**: (35, 49, 60, 65, 451, 476, 518, 688	**10 Sites**: 44, 53, 136, 186, 268, 302, 348, 633, 634, 695	**53 Sites**: 5, 10, 11, 15, 18, 22, 23, 44, 56, 59, 60, 61, 62, 65, 67, 68, 71, 113, 134, 135, 174, 177, 230, 237, 254, 325,378, 422, 427, 442, 451, 453, 467, 479, 633, 634, 635, 636, 639, 657, 680, 682, 687, 690, 713
**FEL:** Pervasive Diversifying Selection (P< 0.05)	**0 Sites**	**2 Sites**: (27, 65)	**2 Sites:** 54, 268	**14 Sites:**10, 22, 23, 62, 67, 230, 363, 392, 427, 442, 451, 473, 512, 656
**PAML** (M8 BEB, No *= PP=90, *PP >95, **PP>95)	**5 Sites:** 13L, 46L, 61A, 61R*, 511A*	**17 Sites:** 5H, 17T, 27V, 35P, 36L*, 65R*, 85G, 172C*, 219Q, 315Y*, 420G, 429C, 450H, 476T*, 498Y*, 589M*, 603Q*	**6 Sites:** 38A, 171V, 213R*, 306R, 391H*, 406C**)	**42 Sites:** 4P, 22 I**, 45A, 46A**, 48M, 52E**, 53Q**, 55K, 56V, 65G*, 67H*, 71G*, 78F, 81T, 129F, 130I, 152L*, 184T*, 192V, 213*, 221A, 230S, 264*, 270R*, 288S, 289C*, 345F, 399S, 403C**, 405R*, 406F, 438W**, 442P*, 448F*, 451C*, 455C*,467S, 473L*, 496R*, 511E*, 512C*, 656F
**FEL:** Purifying Selection (P< 0.05)	**34 sites:** 3, 50, 51, 54, 55, 56, 107, 135, 146, 159, 166, 232, 235, 277, 291, 294, 295, 298, 310, 323, 330, 331, 359, 361, 371, 390, 463, 465, 523, 526, 530, 567, 583, 603	**38 Sites** 37,38,45,78,91, 130,159,163,182,203,204,212,149,252,264,273, 280,292,296,328,330,373,398,402,412,436,447, 461,469,479,522,526,530,535, 555,617,634,714	**37 Sites** 8,12,34,35,58,62,71,76,83,94,153,160,178,198,208,211,239,246,262,273,326,331,369,404,412,444,452,453,486,517,558,564,585,586,637,659,664	**100 Sites:** 2, 8, 9, 51, 86, 90, 93, 97, 98, 101, 103, 105, 111, 121, 138, 140, 143, 155, 156, 158, 165, 170, 185, 194, 198, 205,210,236,240,241,244,245,246,258, 274,285,287,292,293,295,300,303,304, 305,306,318,321,323,324,326,327,329, 330,331,335,337,338,357,358,371,379, 389,425,464,484,499,514,518,522,523, 534,525,541,548,569,570,575,591,595, 598,605,606,610,611,612,615,616,620, 622,624,625,626,637,638,654,665,674, 679,695,707
**FUBAR:** Pervasive Purifying Selection (P< 90)	**28 Sites:** 3, 50, 51, 54, 55, 56, 60, 107, 135, 146, 159, 166, 232, 235, 277, 294, 310, 325, 330, 359, 390, 465, 523, 526, 530, 567, 583, 603	**31 Sites:** 37, 38, 45, 78, 91, 130, 159, 182, 203, 212, 253, 264, 273, 292, 296, 328, 330, 398, 412, 447, 461, 469, 479, 522, 526, 530, 535, 555, 617, 634	**19 Sites:** 34, 58, 62, 76, 178, 208, 239, 262, 273, 326, 404, 412, 444, 452, 486, 517, 586, 659, 664	**107 Sites:** 1, 2, 9, 86, 90, 93, 94, 98, 101, 103, 105, 109, 115, 138, 140, 143, 155, 156, 158, 165, 170, 185, 194, 198, 201, 205, 209, 210, 236, 238, 240, 241, 243, 244, 246, 258, 274, 276, 280, 281, 282, 283, 285, 287, 293, 295, 304, 306, 318, 323, 324, 326, 329, 330, 331, 335, 337, 338, 354, 356, 358, 364, 371, 465, 470, 481, 514, 518, 523, 524, 525, 527, 533, 537, 548, 563, 565, 570, 575, 585, 591, 592, 595, 598, 605, 606, 610, 612, 613, 614, 615, 616, 620, 621, 622, 624, 625, 626, 637, 638, 650, 663, 665, 674, 693, 695, 707

Supplementary Table 3 – Number of negatively selected (NSS) and positively selected (PSS) sites with P<0.05 using FEL and FUBAR analysis.

		Muridae	Cricetidae	Other rodents
FEL (P<0.05)	Negatively selected sites (NSS)	4	87	48
	Positively selected sites (PSS)	14	14	15
FUBAR (PP >90)	Negatively selected sites (NSS)	7	14	28
	Positively selected sites (PSS)	0	3	0

Supplementary Figure 2– House mice express a Type 3 (555-aa) FAM161A isoform in the testes.

a) Various annotated FAM161A isoforms found in house mouse testes, based on transcriptome sequencing performed by Green et al 2018 [4]. **b–c**) FAM161A isoform types 1–3 with exons numbered and approximate locations of forward, reverse (filled, colored triangles), and sequencing primers (open, colored triangles) chosen for PCR with reverse-transcribed mRNA isolated from either house mouse testis or eye tissue (b); and electrophoresis gel with expected sizes (in base pairs) of bands produced using the indicated primers (**c**) "–", no band was expected for that reaction. A cDNA library was constructed using a 3' RACE kit (Roche). The First PCR was performed using the cDNA library, the product of which was used to perform the Second PCR. Sequencing was performed on the Second PCR product following gel extraction. MW, molecular weight. Results were consistent across three independent experiments. **d**) The NCBI house mouse testis EST database showed that eight FAM161A transcripts have the unique C-terminus sequence characteristic of type 3.

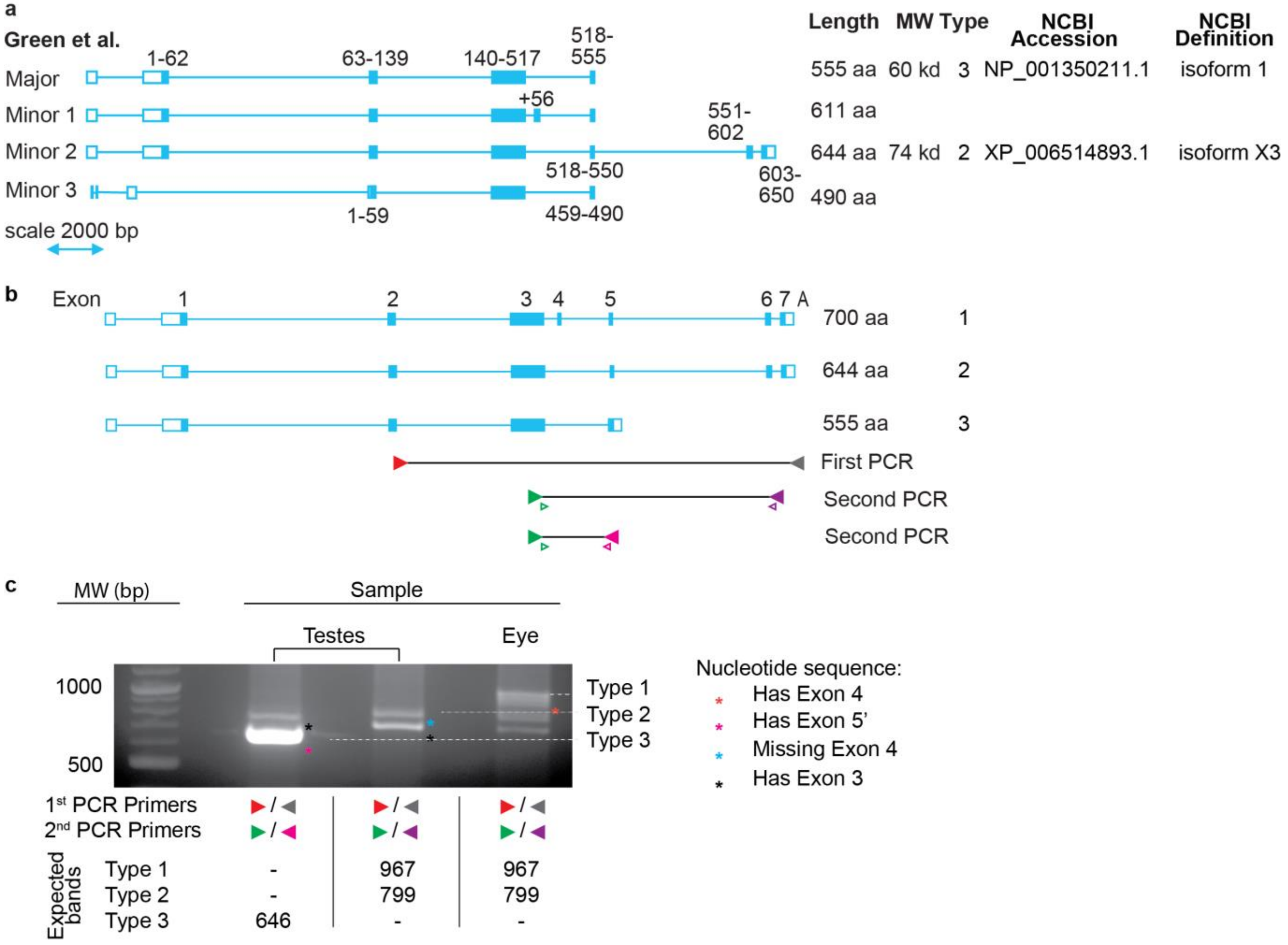

d The NCBI mouse testis EST database shows that eight FAM161A transcripts have the unique, C-terminus sequence characteristic of type 3:

AV283297.2, AI613936.1, AV262776.1, AA492939.1, BB015036.1, AV257583.1, AV283863.1, AA064510.1.

Supplementary Figure 3 – House mouse and human FAM161A isoforms can localize to canonical centrioles in U2OS cells.

a and c) Human FAM161A (hFAM161A) type 2, house mouse FAM161A (mFAM161A) type 2, and mFAM161A type 3 were localized to the centrosome based on pericentrin (**a**) and γ-tubulin (**c**) labeling in U2OS cells. Human FAM161A type 2 and mFAM161A type 2 also exhibit pericentrosomal localization, probably because these isoforms colocalize with microtubules emanating from the centrosome. **b and d**) Quantification showing the percentage of centrioles exhibiting centrosomal, pericentrosomal, and non-centrosomal localization of hFAM161A type 2, mFAM161A type 2, and mFAM161A type 3. All experiments were repeated three times with consistent results. Statistical analyses used are unpaired, two-tailed *t* test. Source data are provided at the Source Data File.

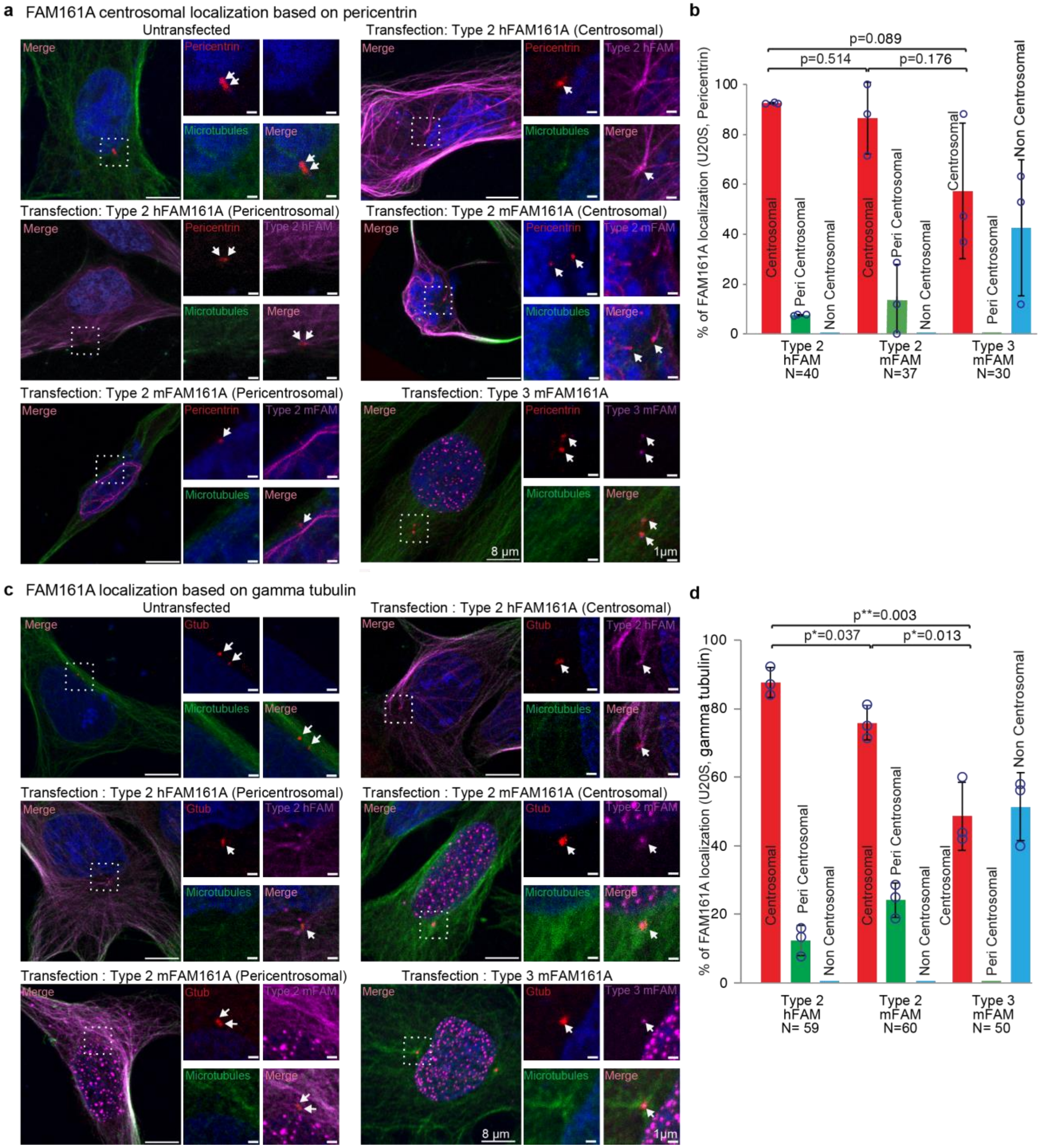

Supplementary Figure 4 – House mouse and human FAM161A isoforms can localize to canonical centrioles in 3T3 cells.

a and c) Human FAM161A (hFAM161A) type 2, house mouse FAM161A (mFAM161A) type 2, and mFAM161A type 3 were localized to the centrosome based on pericentrin (**a**) and γ-tubulin (**c**) labeling in 3T3 cells. Human FAM161A type 2 and mFAM161A type 2 also exhibit pericentrosomal localization, probably because these isoforms colocalize with microtubules emanating from the centrosome. **b and d**) Quantification showing the percentage of centrioles exhibiting centrosomal, pericentrosomal, and non-centrosomal localization of hFAM161A type 2, mFAM161A type 2, and mFAM161A type 3. All experiments were repeated three times with consistent results. Statistical analyses used are unpaired, two-tailed *t* test. Source data are provided at the Source Data File.

a FAM161A localization based on pericentrin

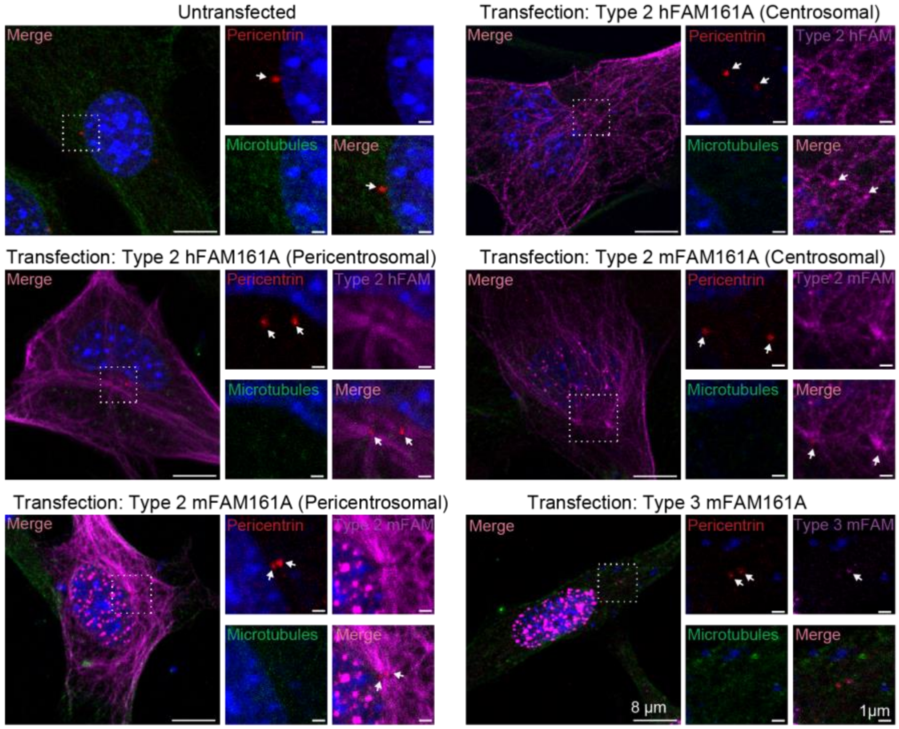

b

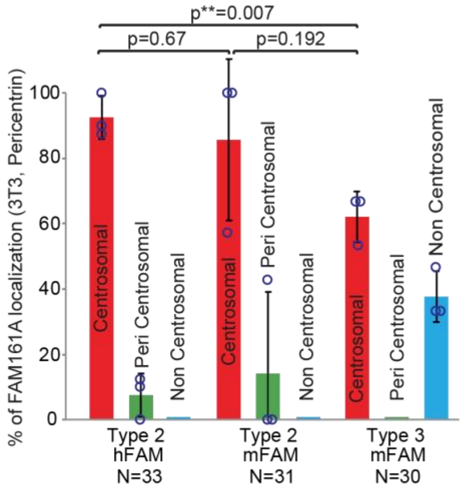

c FAM161A localizaion based on gamma tubulin

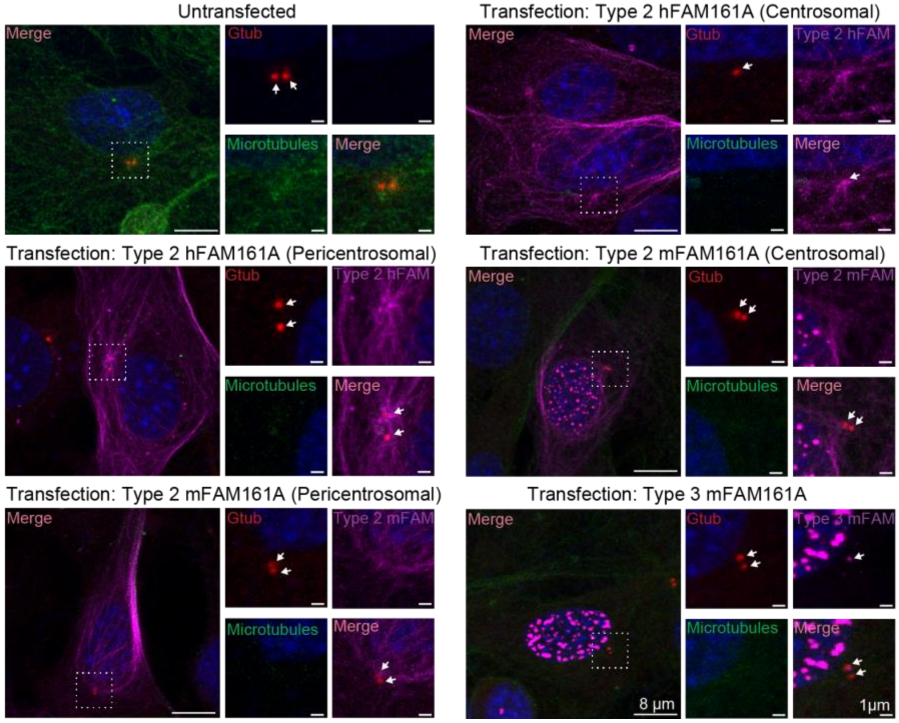

d

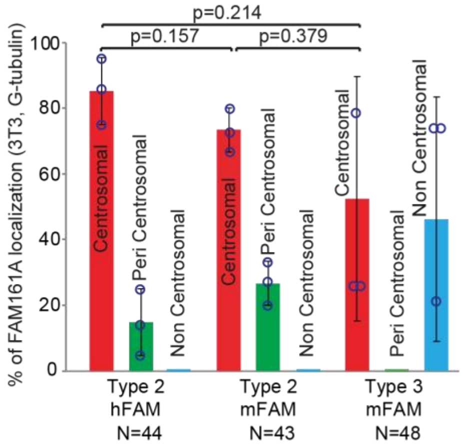

Supplementary Figure 5 – The mechanism of rod protein interaction

a) Expression of human FAM161A (hFAM161A) type 2, human POC1B (hPOC1B), and human POC5 (hPOC5) and quantification of their colocalization with tubulin in U2OS cells. **b**) Expression of various fragments of hFAM161A type 2 with full-length hPOC1B and quantification of FAM161A colocalization with hPOC1B. **c**) Expression of various fragments of hFAM161A type 2 with hPOC5 and quantification of FAM161A colocalization with hPOC5. Scale bars are 10 µm. **d**) Yeast two-hybrid analysis to map the hPOC5-interacting domain of hFAM161A. BD, binding domain; AD, activation domain; DDO, double dropout (-Leu, -Trp); TDO, triple dropout (-Leu, -Trp, -His); QDO, quadruple dropout (-Leu, -Trp, -His, -Ade). **e**) Yeast two-hybrid analysis to map the domain mediating the interaction between hPOC1B and hPOC5. **f**) Model depicting the mechanism of interaction between rod proteins. WD, WD40 repeat domains; CBR, centrin-binding region; EF-hand, a calcium-binding motif. CC, coiled-coil domain; n, number of cells. The data shown are the representative images and quantification from three independent experiments. The graphs are presented as box and whisker plots, where upper and lower bounds show interquartile range, line within the box shows median, and the whiskers show minimum and maximum data points. ****P<0.0001, ***P<0.001, **P<0.01, *P<0.05 (unpaired, two-tailed *t-test*, exact p-values are provided in the source data file), ns, not significant; n= number of transfected cells, BD, Binding Domain; AD, Activation Domain. Source data are provided in the Source Data File.

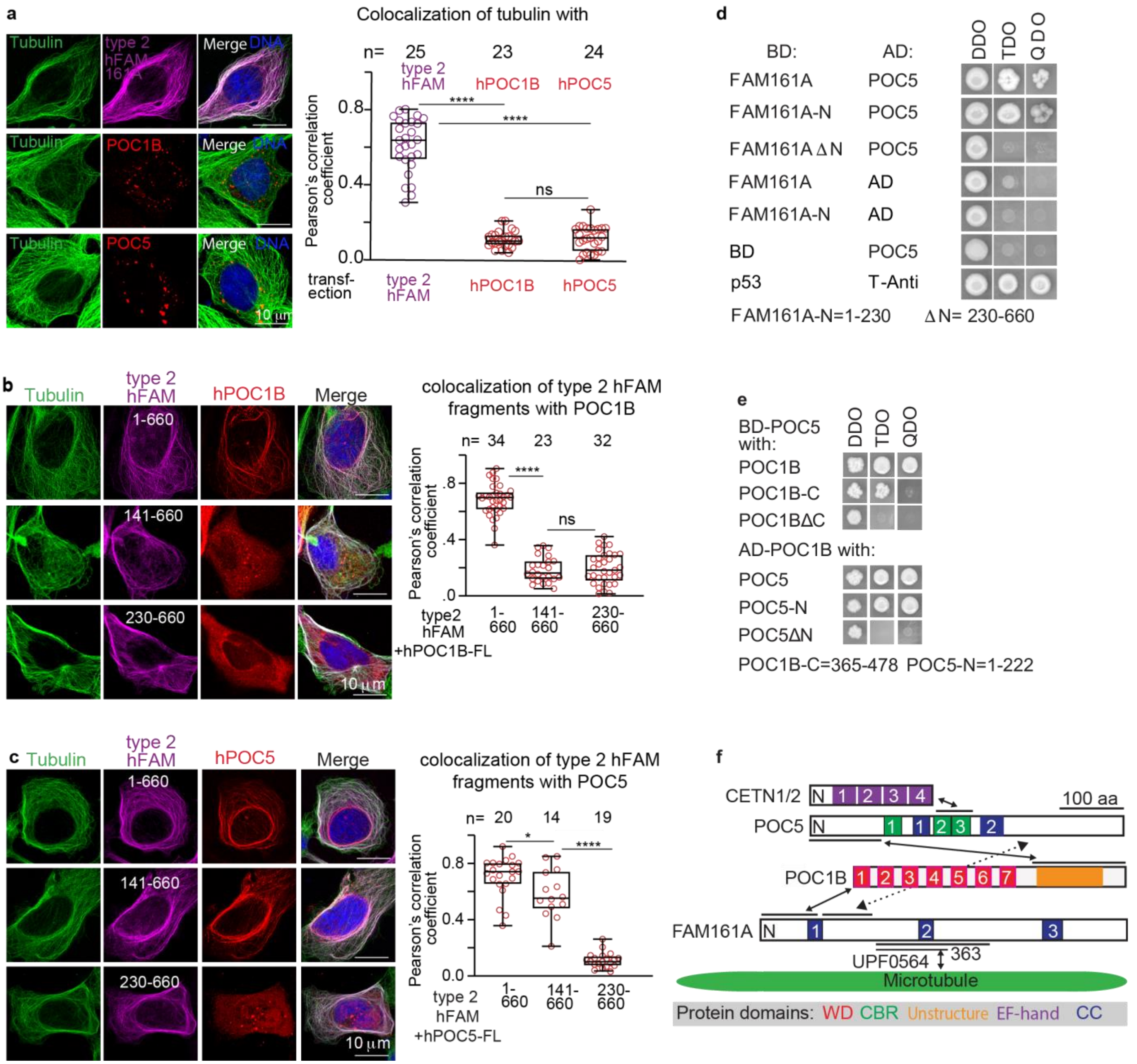
a
Tubulin
type 2 hFAM 161A
Merge DNA
POC1B
POC5
10 µm
Colocalization of tubulin with
n= 25 23 24
type 2 hFAM
hPOC1B
hPOC5
Pearson's correlation coefficient
0.8
0.4
0.0
****
ns
transf-ection
b
Tubulin
type 2 hFAM
hPOC1B
Merge
1-660
141-660
230-660
colocalization of type 2 hFAM fragments with POC1B
n= 34 23 32
type2 hFAM +hPOC1B-FL
1-660
141-660
230-660
c
hPOC5
colocalization of type 2 hFAM fragments with POC5
n= 20 14 19
*
type 2 hFAM +hPOC5-FL
d
BD:
AD:
DDO
TDO
QDO
FAM161A
POC5
FAM161A-N
FAM161A ΔN
AD
BD
p53
T-Anti
FAM161A-N=1-230
ΔN= 230-660
e
BD-POC5 with:
POC1B
POC1B-C
POC1BΔC
AD-POC1B with:
POC5-N
POC5ΔN
POC1B-C=365-478 POC5-N=1-222
f
CETN1/2
100 aa
UPF0564
363
Microtubule
Protein domains: WD CBR Unstructure EF-hand CC

Supplementary Figure 6 – FAM161A isoforms exhibit different localization patterns in 3T3 cells.

a) Expression analysis of human FAM161A (hFAM161A) type 2 (second panel from left), house mouse FAM161A (mFAM161A) type 2 (middle four panels), mFAM161A type 3 (right panel) in 3T3 cells. **b**) Quantification showing the percentage of cells exhibiting each of the various expression patterns observed during expression analysis of hFAM161A type 2, mFAM161A type 2, and mFAM161A type 3. C, “Cytoplasmic”; I, “Intranuclear”; I+C, “Intranuclear + Cytoplasmic”; P, “Perinuclear”. The experiment was repeated three times with consistent results. Source data are provided at the Source Data File.

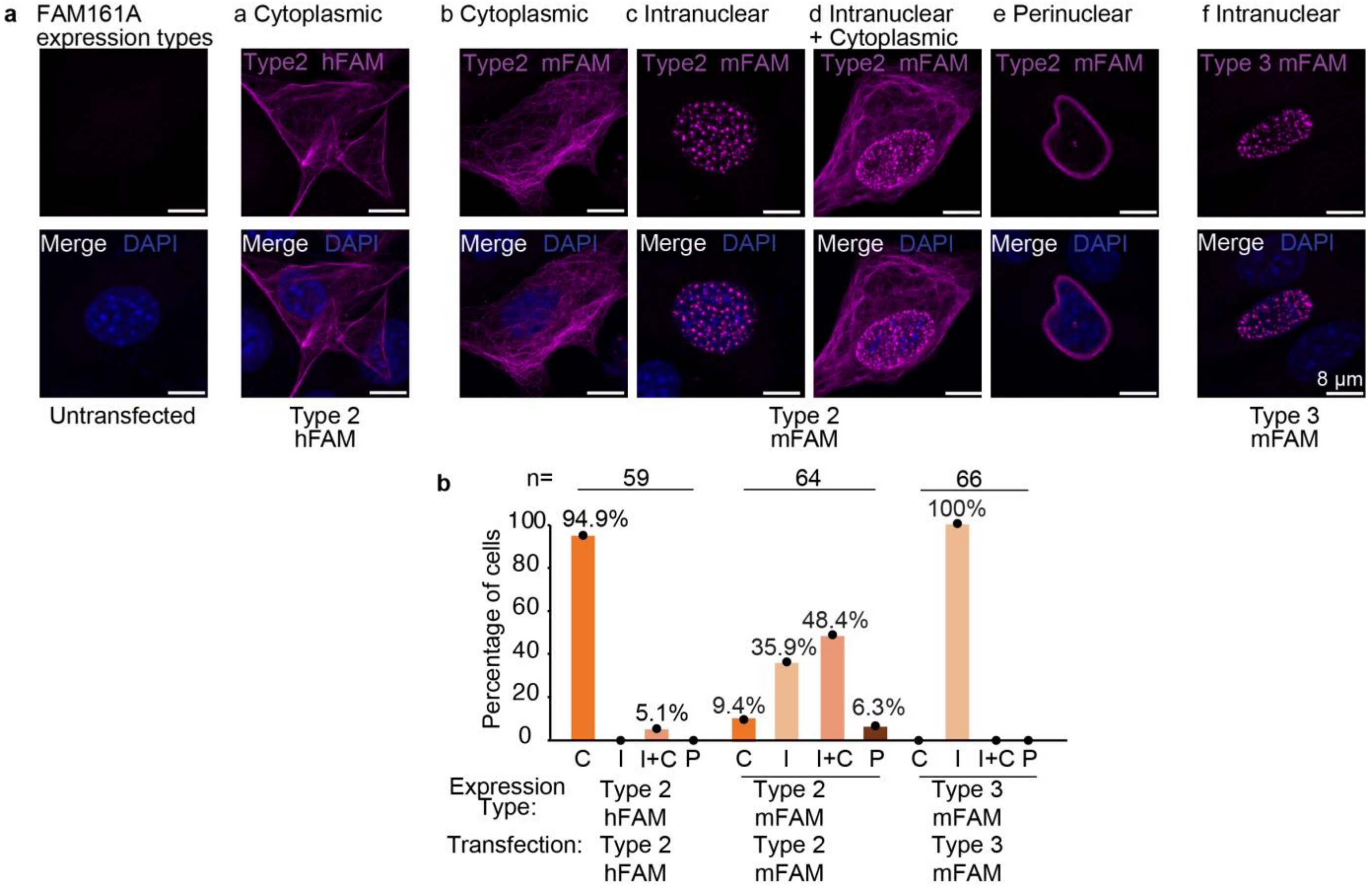

Supplementary Figure 7– FAM161A sequence alignment

Alignment of the amino acid sequences of human FAM161A type 1 (Hs1), human FAM161A type 2 (Hs2), mouse FAM161A type 1 (Mm1), mouse FAM161A type 2 (Mm2), and mouse FAM161A type 3 (Mm3). The various protein domains are marked using color coding.

```
Hs1     MATSHRVAKLVASSLQTPVNPITGARVAQYEREDPLKALAAAEAILEDEEEE--KVAQPA 58
Hs2     MATSHRVAKLVASSLQTPVNPITGARVAQYEREDPLKALAAAEAILEDEEEE--KVAQPA 58
Mm1     MASPHQEARLAAASLLLSANPITGARVAQYESEDSLESLVAAGLAAAMEEEEQKKVWQMR 60    POC1B Binding segment
Mm2     MASPHQEARLAAASLLLSANPITGARVAQYESEDSLESLVAAGLAAAMEEEEQKKVWQMR 60
Mm3     MASPHQEARLAAASLLLSANPITGARVAQYESEDSLESLVAAGLAAAMEEEEQKKVWQMR 60
        **: *: *:*.*:**   .************ ** *::*.**       :**::** *

Hs1     GASADLNTSFSGVDEHAPISYEDFVNFPDIHHSNEEYFKKVEELKAAHIETMAKLEKMYQ 118
Hs2     GASADLNTSFSGVDEHAPISYEDFVNFPDIHHSNEEYFKKVEELKAAHIETMAKLEKMYQ 118
Mm1     -ASAGCH-NFSGKDE--EVAFESTMNFSDIYHSDEEYFRKLKDLKAVHEETMSKLEKMYQ 116
Mm2     -ASAGCH-NFSGKDE--EVAFESTMNFSDIYHSDEEYFRKLKDLKAVHEETMSKLEKMYQ 116
Mm3     -ASAGCH-NFSGKDE--EVAFESTMNFSDIYHSDEEYFRKLKDLKAVHEETMSKLEKMYQ 116
        .  :    .*** **   :::*. :** **:**:****:*:::***.* ***:*******

Hs1     DKLHLKEVQPVVIREDSLSDSSRSVSEKNSYHPVSLMTSFSEPDLGQSSSLYVSSSEEEL 178
Hs2     DKLHLKEVQPVVIREDSLSDSSRSVSEKNSYHPVSLMTSFSEPDLGQSSSLYVSSSEEEL 178    POC5 Binding segment
Mm1     DKLNIKDIQAGFIRDGISDSSSSSASEKSCSHPALSVTSLSEPDLDGSSSLST-TTDEGL 175
Mm2     DKLNIKDIQAGFIRDGISDSSSSSASEKSCSHPALSVTSLSEPDLDGSSSLST-TTDEGL 175
Mm3     DKLNIKDIQAGFIRDGISDSSSSSASEKSCSHPALSVTSLSEPDLDGSSSLST-TTDEGL 175
        ***::*::*  .**:.  ..** *.***.. **.  :**:*****. **** . :::* *

Hs1     PNLEKEYPRKNRMMTYAKELINNMWTDFCVEDYIRCKDTGFHAAEKRRKKRKEWVPTITV 238
Hs2     PNLEKEYPRKNRMMTYAKELINNMWTDFCVEDYIRCKDTGFHAAEKRRKKRKEWVPTITV 238
Mm1     PDLEEKTPGESSAMVHAQELINNMWNDFSVEDYIQYDSDS-RTAKKKRKKAKSLTPKITV 234
Mm2     PDLEEKTPGESSAMVHAQELINNMWNDFSVEDYIQYDSDS-RTAKKKRKKAKSLTPKITV 234
Mm3     PDLEEKTPGESSAMVHAQELINNMWNDFSVEDYIQYDSDS-RTAKKKRKKAKSLTPKITV 234
        *:**:: * :.  *.:*:*******.**.*****: .. . ::*:*:*** *. .*.***

Hs1     PEPFQMMIREQKKKEESMKSKSDIEMVHKALKKQEEDPEYKKKFRANPVPASVFLPLYHD 298
Hs2     PEPFQMMIREQKKKEESMKSKSDIEMVHKALKKQEEDPEYKKKFRANPVPASVFLPLYHD 298    dimerization domain
Mm1     PVPFEMTVREQNRREKALSARSDLE--TKLLKRDEDDAECKKKFRANPVPSCVLLPLYED 292
Mm2     PVPFEMTVREQNRREKALSARSDLE--TKLLKRDEDDAECKKKFRANPVPSCVLLPLYED 292
Mm3     PVPFEMTVREQNRREKALSARSDLE--TKLLKRDEDDAECKKKFRANPVPSCVLLPLYED 292
        * **:* :***:::*:::.::**:*   * **::*:* * **********:.*:****.*

Hs1     LVKQKEERRRSLKEKSKEALLASQKPFKFIAREEQKRAAREKQLRDFLKYKKKTNRFKAR 358
Hs2     LVKQKEERRRSLKEKSKEALLASQKPFKFIAREEQKRAAREKQLRDFLKYKKKTNRFKAR 358
Mm1     LVKQSEERRKKARERNRAALLASLKPFKFIAREEQKQAVREKKLRDLFRAKRKTNQFKAK 352
Mm2     LVKQSEERRKKARERNRAALLASLKPFKFIAREEQKQAVREKKLRDLFRAKRKTNQFKAK 352
Mm3     LVKQSEERRKKARERNRAALLASLKPFKFIAREEQKQAVREKKLRDLFRAKRKTNQFKAK 352
        ****.****:. :*:.: ***** ************:*.***:***::: *:***:***:

Hs1     PIPRSTYGSTTNDKLKEEELYRNLRTQLRAQEHLQNSSPLPCRSACGCRNPRCPEQAVKL 418
Hs2     PIPRSTYGSTTNDKLKEEELYRNLRTQLRAQEHLQNSSPLPCRSACGCRNPRCPEQAVKL 418
Mm1     PVPRFIYRPAASDKPKEEELYGDSRMLPKVRDLL-QNSPWPSRSACR--RFRDPRSPAKP 409
Mm2     PVPRFIYRPAASDKPKEEELYGDSRMLPKVRDLL-QNSPWPSRSACR--RFRDPRSPAKP 409
Mm3     PVPRFIYRPAASDKPKEEELYGDSRMLPKVRDLL-QNSPWPSRSACR--RFRDPRSPAKP 409
        *:**  *  ::.** ****** : *   :.:: * :.** *.****   . * *.. .*

Hs1     KCKHKVRCPTPDFEDLPERYQKHLSEHKSPKLLTVCKPFDLHASPHASIKREKILADIEA 478
Hs2     KCKHKVRCPTPDFEDLPERYQKHLSEHKSPKLLTVCKPFDLHASPHASIKREKILADIEA 478    UPF0564
Mm1     RGKHRRRCLRRDG--DLEKWKEPFSEYSFLKCPMLCEECCLHESPCDSDKRQKLLADIRA 467
Mm2     RGKHRRRCLRRDG--DLEKWKEPFSEYSFLKCPMLCEECCLHESPCDSDKRQKLLADIRA 467
Mm3     RGKHRRRCLRRDG--DLEKWKEPFSEYSFLKCPMLCEECCLHESPCDSDKRQKLLADIRA 467
        : **: **   *     *:::: :**:.  *   :*:   ** **  * **:*:****.*

Hs1     DEENLKETRWPYLSPRRKSPVRCAGVNPVPCNCNPPVPTVSSRGREQAVRRSLEEKKMLE 538
Hs2     DEENLKETRWPYLSPRRKSPVRCAGVNPVPCNCNPPVPTVSSRGREQAVR---------- 528
Mm1     DEEILRETRRPGRSPRRKSPGRSSNPKPRPHECSPPMPTASSRGREQAIRKSLEEKKMLE 527    Exon 4
Mm2     DEEILRETRRPGRSPRRKSPGRSSNPKPRPHECSPPMPTASSRGREQAIR---------- 517
Mm3     DEEILRETRRPGRSPRRKSPGRSSNPKPRPHECSPPMPTASSRGREQAIR---------- 517
        *** *:*** *  ******* *.:. :* * :*.**:**.********:*

Hs1     EERNRILTKQKQRMKELQKLLTTRAKAYDSHQSLAQISKSRVKCLRKSEKERMREYQREL 598
Hs2     ----------------------------------------------KSEKERMREYQREL 542
Mm1     EERNRILTKQKQRMKDLQKLLATRVKAYGSHQSLSQIVKARVKELRRSEKARMREYWQEL 587
Mm2     ----------------------------------------------RSEKARMREYWQEL 531
Mm3     ----------------------------------------------RSEKARMREYWQEL 531
                                                      :*** ***** :**

Hs1     EEREEKLKKRPLLFERVAQKNARMAAEKHYSNTLKALGISDEFVSKKGQSGKVLEYFNNQ 658
Hs2     EEREEKLKKRPLLFERVAQKNARMAAEKHYSNTLKALGISDEFVSKKGQSGKVLEYFNNQ 602
Mm1     EEQEEKLQKRPMLFERVTQRNARMAAERCYSNTLKALGLLEEFVSEKGQSGKVSEDFTRQ 647
Mm2     EEQEEKLQKRPMLFERVTQRNARMAAERCYSNTLKALGLLEEFVSEKGQSGKVSEDFTRQ 591
Mm3     EEQEEKLQKRPMLFERVTQVVFIG------------------------------------ 555    Type 3 unique sequence
        **:****:***:*****:*

Hs1     ETKSVTEDKESFNEEEKIEERENGEENYFIDTNSQDSYKEKDEANEES--EEEKSVEESH 716
Hs2     ETKSVTEDKESFNEEEKIEERENGEENYFIDTNSQDSYKEKDEANEES--EEEKSVEESH 660
Mm1     ELRSCTLEKESSYG----EERENEEESYLTDISSQDSCKGNKEDIKDIKSGEENSGE--- 700
Mm2     ELRSCTLEKESSYG----EERENEEESYLTDISSQDSCKGNKEDIKDIKSGEENSGE--- 644
Mm3     ------------------------------------------------------------ 555
```

Supplementary Figure 8 – POC5 localizes to canonical centrioles when each of the three FAM161A isoforms are overexpressed.

a) POC5 localization relative to overexpressed human FAM161A (hFAM161A) type 2, house mouse FAM161A (mFAM161A) type 2, and mFAM161A type 3 in U2OS cells. Human FAM161A type 2 and mFAM161A type 2 exhibit centrosomal and pericentrosomal localization. **b**) Quantification showing the percentage of centrioles exhibiting centrosomal, pericentrosomal, and non-centrosomal localization of hFAM161A type 2, mFAM161A type 2, and mFAM161A type 3. **c**) POC5 localization relative to astral microtubules when overexpressing hFAM161A type 2, mFAM161A type 2, and mFAM161A type 3 in U2OS cells. **d**) Quantification showing the percentage of cells exhibiting centrosomal localization of POC5 based on astral microtubules. All experiments were repeated three times with consistent results. Source data are provided at the Source Data File.

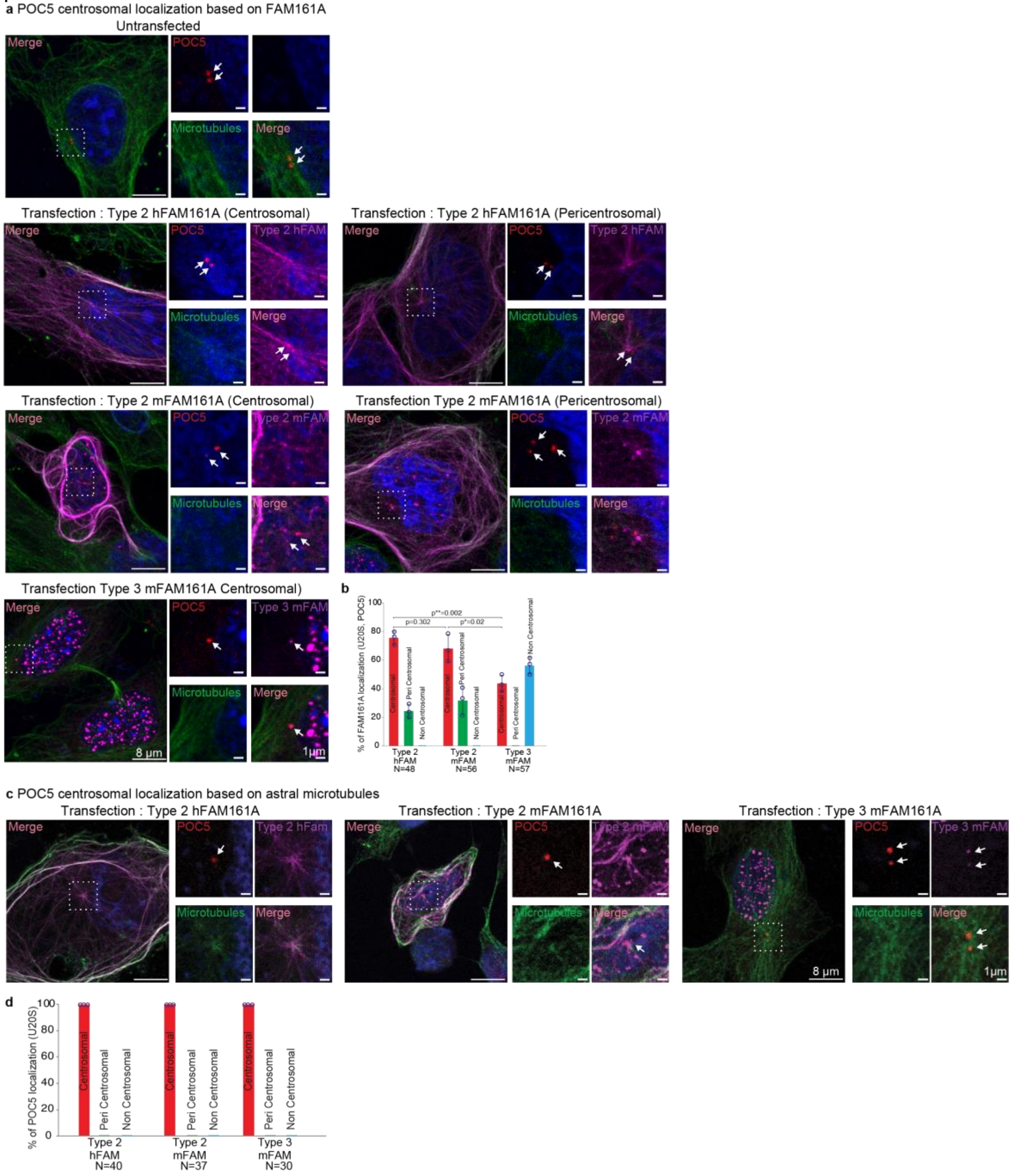

Supplementary Figure 9 – House mouse FAM161A type 3 inhibits type 2 from recruiting POC1B to the microtubules.

a–c) Expression of human POC1B (hPOC1B) with house mouse FAM161A (mFAM161A) type 2 (**a**), mFAM161A type 3 (**b**), and both mFAM161A types 2 & 3 (**c**). Only one FAM161A isoform at a time is shown in panel C; however, the cells were transfected with all three proteins (i.e., hPOC1B and mFAM161A types 2 & 3). Scale bars are 8 µm. **d**) Quantification of hPOC1B colocalization with tubulin under various transfection conditions. **e**) mFAM161A type 3 and hPOC1B colocalize in extranuclear foci. Shown in inset. The graphs are presented as box and whisker plots, where upper and lower bounds show interquartile range, the line within the box shows median, and the whiskers show minimum and maximum data points. ****P<0.0001(unpaired, two tailed *t* test, exact p-values are provided in source data file). n, number of cells. Scale bars are 5 µm. All images are representative of three independent experiments. The quantification data was compiled from three independent experiments. Source data are provided at the Source Data File.

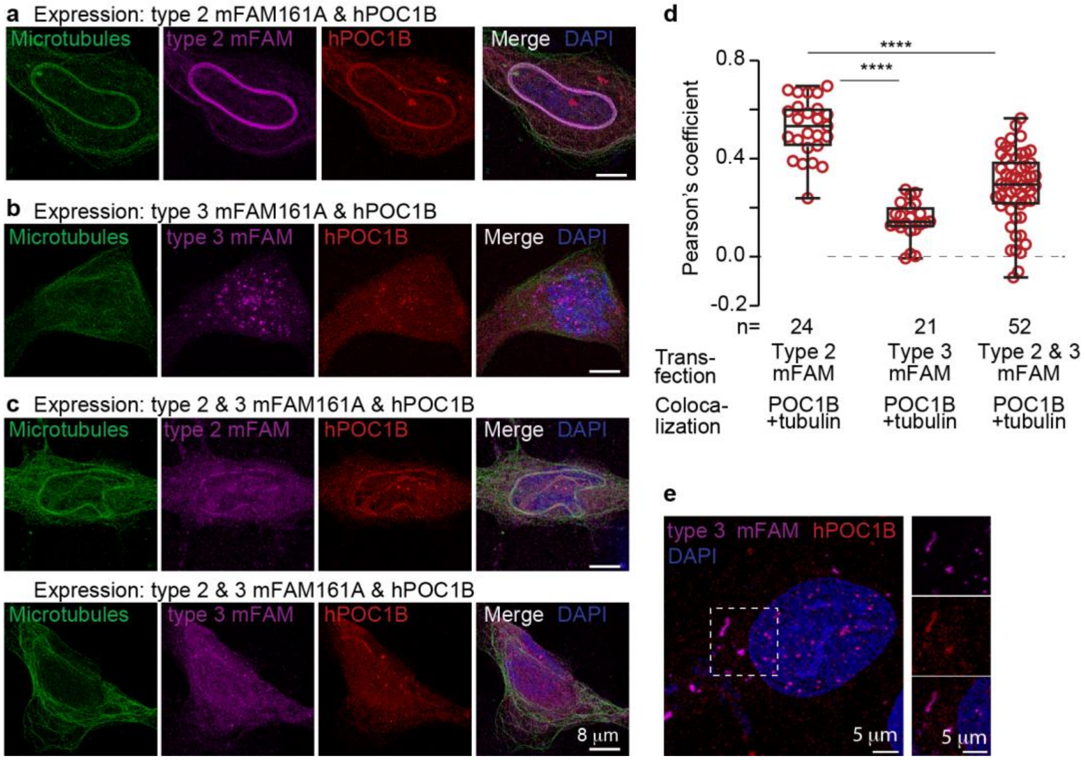

Supplementary Figure 10 – FAM161A labels the proximal and distal centrioles of dog spermatozoa.

Confocal (deconvolution) microscopy of the dog distal centriole showed that FAM161A exhibits prominent left- and right-side labeling, suggesting that the distal centriolar rods are far away from each other. PC, proximal centriole; DC, distal centriole; Ax, axoneme. Scale bars are 1 µm. All images are representative of at least two independent experiments.

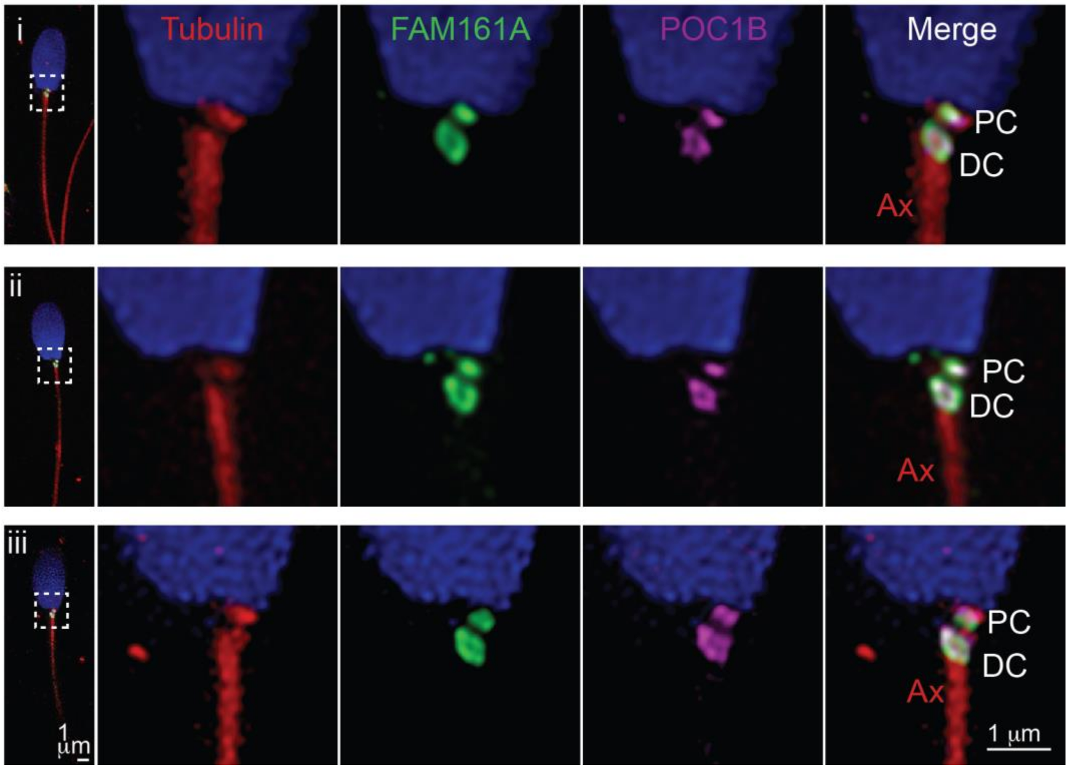

Supplementary Figure 11 – Centriole remodeling generates species-specific centriolar sizes.

Quantification of centriolar length based on CETN1 labeling in bovine (Bt), rabbit (Oc), and human (Hs) testes at spermatogonia/spermatocyte (Sg) and round spermatid (RS) cell stages. The graphs are presented as box and whisker plots, where upper and lower bounds show interquartile range, line within the box shows median, and the whiskers show minimum and maximum data points. C1/2, centriole 1/2; PC, proximal centriole; DC, distal centriole. ****P<0.0001, **P<0.01(unpaired, two tailed *t* test, exact p-values are provided in source data file); ns, not significant; n, number of centrioles. The result is based on immunostaining with CETN1 and confocal imaging. The data was accumulated over three independent experiments. Source data are provided at the Source Data File.

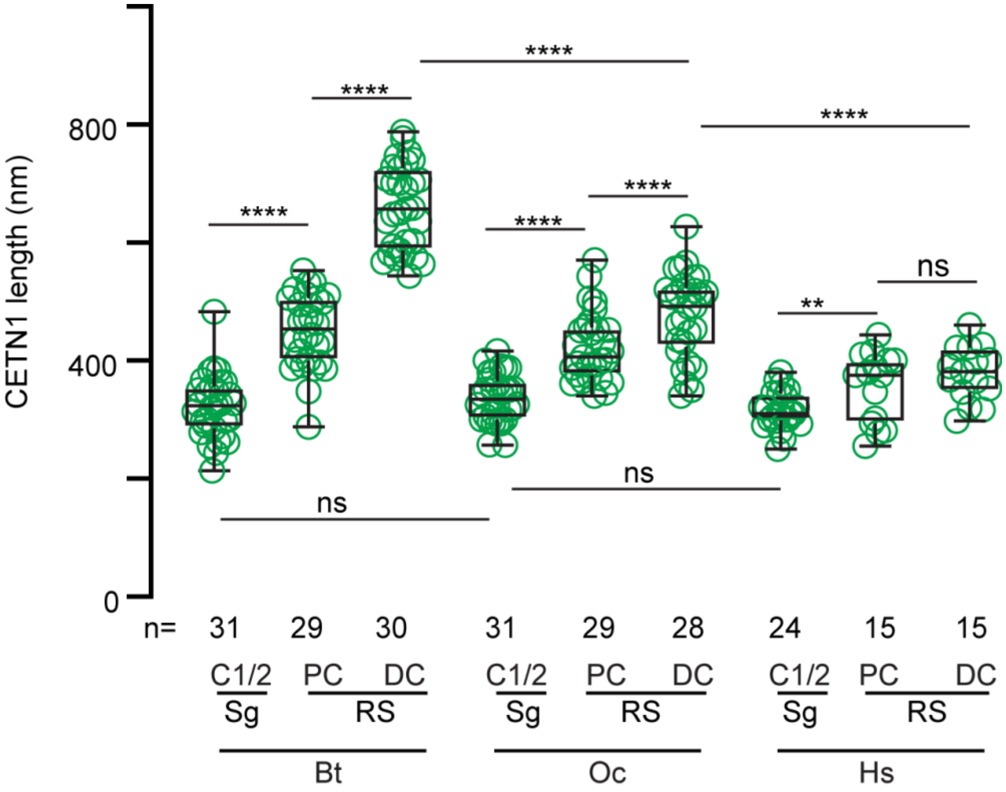

Supplementary Figure 12 – Quantification in the proximal and distal centrioles shows localization intensity changes similar to that observed during total centriole quantification.

Quantification of various proteins in individual centrioles (C1 and C2) of spermatogonia/spermatocytes (Sg), and in proximal and distal centrioles (PC and DC) of round (RS) and elongated spermatids (ES) in bovine. The graphs are presented as box and whisker plots, where upper and lower bounds show interquartile range, line within the box shows median, and the whiskers show minimum and maximum data points. ****P<0.0001, ***P<0.001, **P<0.01, *P<0.05 (unpaired, two tailed *t* test, exact p-values are provided in source data file); ns, not significant; n, number of cells. Source data are provided at the Source Data File.

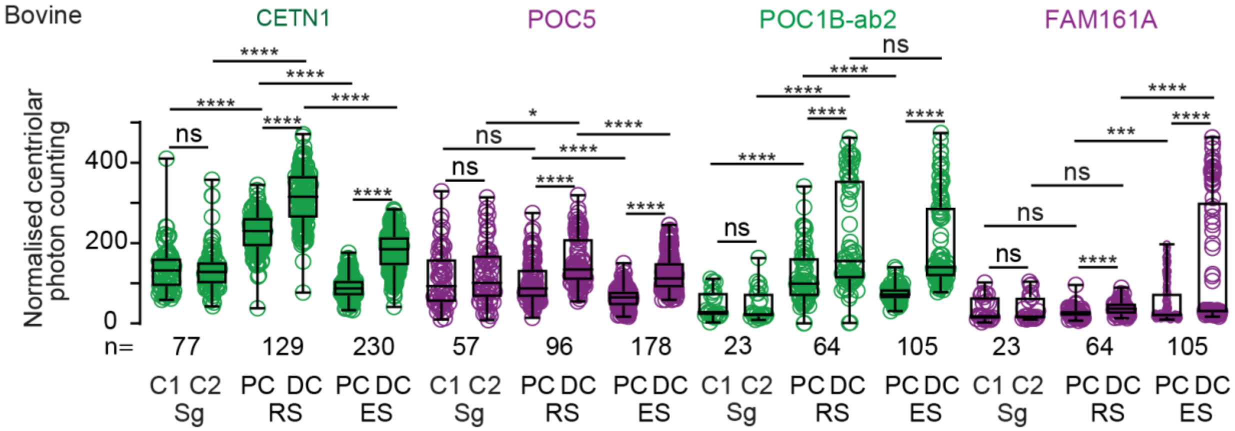

Supplementary Figure 13 – The bovine distal centriole begins splaying in round spermatids and splits in elongated spermatids.

a) 3D-STORM imaging of bovine testes with rod protein staining during spermatogenesis. The white arrow marks the splayed centriole. **b**) Centriolar length in spermatogonia/spermatocytes (Sg), round spermatids (RS), and elongated spermatids (ES). The C1/2 panel includes both C1 and C2. **c**) Proximal and distal centriolar width at their caudal (aka tip) and rostral (aka base) ends in round spermatids. C1/2, centrioles 1 and 2; PC, proximal centriole; DC, distal centriole. **d**) Distal centriolar rod length and width measurements based on POC5 staining in elongated spermatids. All data shown were accumulated over three independent experiments. The graphs are presented as box and whisker plots, where upper and lower bounds show interquartile range, line within the box shows median, and the whiskers show minimum and maximum data points. Data shown in **d** is an average ± SD. ****P<0.0001, ***P<0.001, **P<0.01, *P<0.05 (unpaired, two tailed *t* test, exact p-values are provided in source data file); ns, not significant; n, number of centrioles. Source data are provided at the Source Data File

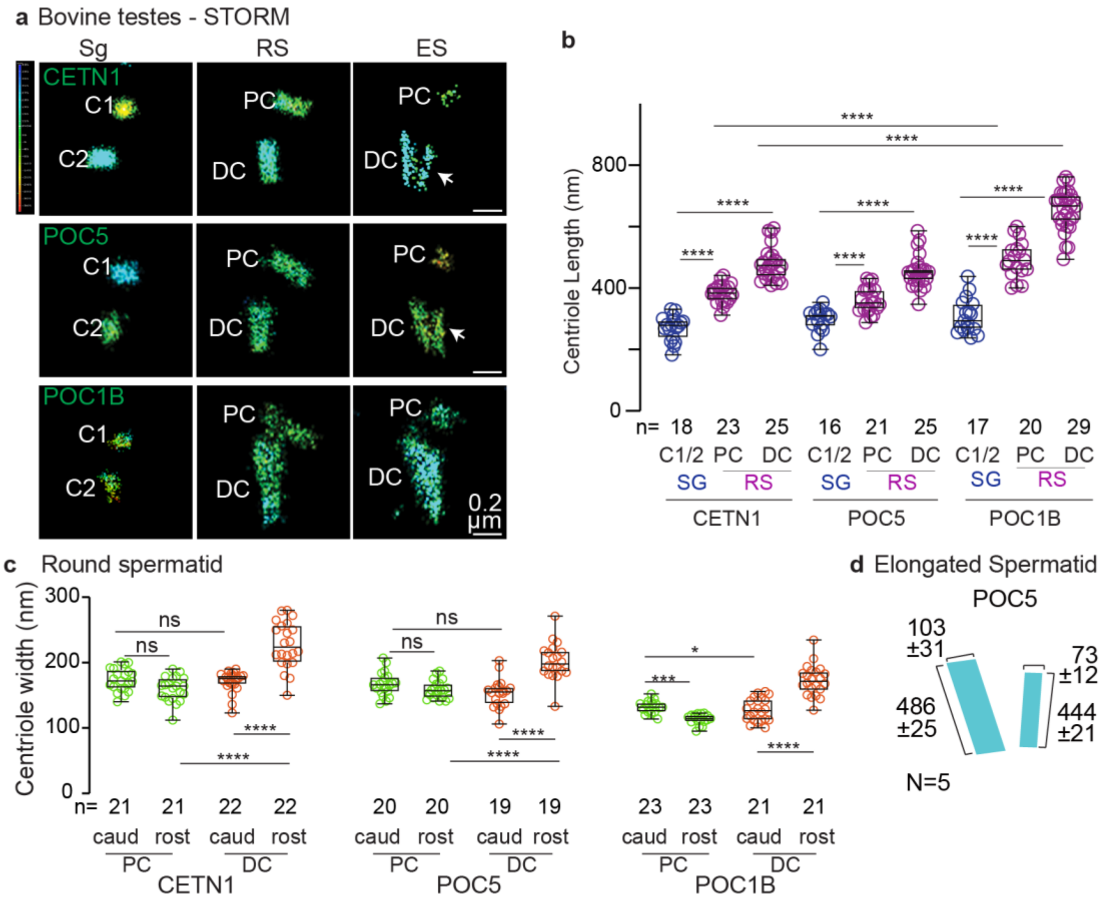

Supplementary Figure 14 – FAM161A N-terminus Ab labeling in mouse testes

Immunostaining using FAM161A N-terminus antibody and POC1B ab2 in house mouse round spermatids (RS). PC, proximal centriole; DC, distal centriole. Scale bars are 1 µm. The result was consistent in three independent immunostainings.

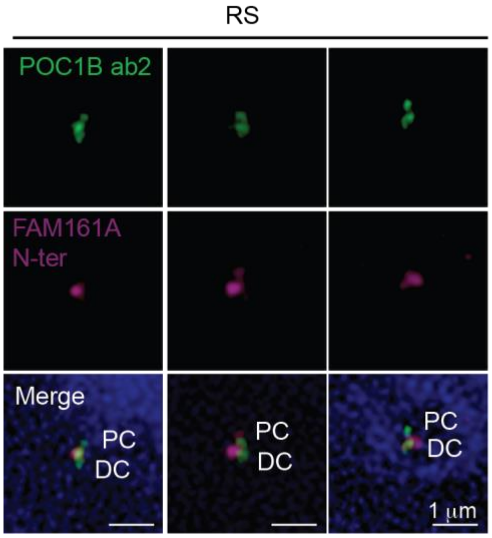

Supplementary Figure 15 – Quantification in the proximal and distal centrioles shows localization intensity changes similar to that observed during total centriole quantification.

Quantification of various proteins in individual centrioles (C1 and C2) of spermatogonia/spermatocytes (Sg), and in proximal and distal centrioles (PC and DC) of round (RS) and elongated spermatids (ES) in mice. The distal centriole in the round spermatid was determined as the larger and brighter POC5-labeled centriole. The graphs are presented as box and whisker plots, where upper and lower bounds show interquartile range, line within the box shows median, and the whiskers show minimum and maximum data points. ****P<0.0001, ***P<0.001, **P<0.01, *P<0.05 (unpaired, two-tailed *t-test*, exact p-values are provided in source data file); ns, not significant; n, number of cells. Source data are provided in the Source Data File.

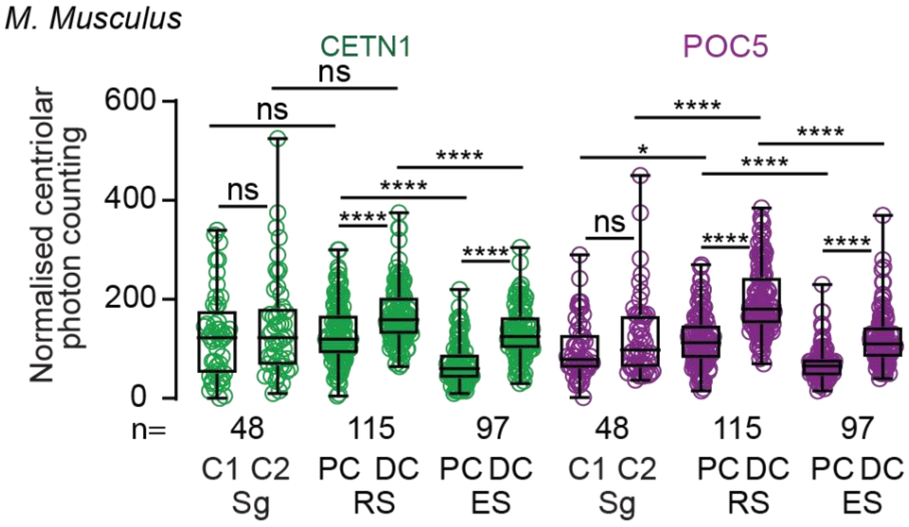

Supplementary Table 4 – Antibodies used in this study.

Antibody (Production animal)	Company (Catalog number)	Concentration (Application)
POC5 ab1 (Rabbit)	Gifted by Dr. Bernan (PMID #19349582)	1:200 (Confocal)<br>1:100 (STORM)
POC5 ab2 (Rabbit)	Thermo Fisher Scientific (PA5-24308)	1:200 (Confocal)<br>1:100 (STORM)
CETN1 (Mouse)	Santa Cruz (2A6)	1:10 (Confocal)<br>1:5 (STORM)
POC1B ab1 (Rabbit)	Thermo Fisher Scientific (PA5-24495)	1:300 (Confocal)
POC1B ab2 (Mouse)	Thermo Fisher Scientific (H00282809-B01P)	1:300 (Confocal)<br>1:100 (STORM)
FAM161A ab1 (Rabbit)	Sigma Aldrich (HPA032119)	1:300 (Confocal)
FAM161A ab2 (Rabbit)	Novus Biologicals (NBP1-91508)	1:1000 (Western)
FAM161A N-terminus (Rabbit)	Gifted by Dr. Dror Sharon (Hadassah-Hebrew University Medical Center)	1:200 (Confocal)
Tubulin (Mouse)	DSHB (Developmental Studies Hybridoma Bank) (E7)	1:20 (Supernatant)<br>1:100 (Concentrate)
Tubulin (Sheep)	Cytoskeleton, Inc. (ATN02)	1:500 (Confocal)
Anti-HA (Rabbit)	Invitrogen (SG77)	1:200 (Confocal)<br>1:1000 (Western)
Anti-FLAG (Mouse)	Invitrogen (FG4R)	1:400 (Confocal)<br>1:1000 (Western)
Anti-Rabbit A647 (Donkey)	Jackson ImmunoResearch (711-605-152)	1:300 (Confocal)<br>1:100 (STORM)
Anti-Mouse A647 (Donkey)	Jackson ImmunoResearch (715-605-150)	1:300 (Confocal)<br>1:100 (STORM)
Anti-Rabbit A488 (Donkey)	Jackson ImmunoResearch (711-545-152)	1:200 (Confocal)
Anti-Sheep A555 (Donkey)	Thermo Fisher Scientific (A-21436)	1:500 (Confocal)
Anti-Mouse A488 (Donkey)	Jackson ImmunoResearch (715-545-150)	1:200 (Confocal)
Anti-Sheep A488 (Donkey)	Jackson ImmunoResearch (715-545-003)	1:300 (Confocal)

Supplementary Table 5 – Primers used in this study.

Application	Primer	Sequence
3’ RACE	Oligo d(T)-Anchor	5’-GACCACGCGTATCGATGTCGACTTTTTTTTTTTTTTTTV-3’<br>*Mlu* | site *Cla* | site *Sal* | site, V = A, C, or G (what is that)
1st PCR	2FW	5’-CAGGAAACTCAAAGACCTGAAGG-3’
	PCR Anchor	5’-GACCACGCGTATCGATGTCGAC-3’
2nd PCR	3FW	5’-GCCAGCTGCGAGTGACAAGC-3’
	5RV	5’-CTGGAGGAACAAGAGGAAAAACTGC-3’
	5*RV	5’-GCCTCCACCTTTGTGCTCCGTC-3’
	7RV	5’-GAACCACAGCTGACACAAATGG-3’
Sequencing	Seq. 3FW	5’-GCAGACATCAGAGCAGATGAAG-3’
	Seq. 5RV	5’-GGAGTGAAAAGGCCAGGATGAG-3’
	Seq. 5*RV	5’-CCAGCTTTCTGTTTCTCAGTAC-3’
	Seq. 7RV	5’-GGAGAAGAAAGAGAGAATGAGG-3’

Supplementary Table 6 – Species analyzed in this study.

a. Rodent sequences analyzed in this study.

Organism	Length of amino acid sequence	Accession ID
**Mus musculus**<br>house mouse	**700**	XM_006514828.5
**Rattus norvegicus**<br>Norway rat	**653**	XM_017599131.2
**Heterocephalus glaber**<br>naked mole-rat	**706**	XM_004867228.3
**Nannospalax galili**<br>Upper Galilee Mountains blind mole rat	**709**	XM_017800967.2
**Mesocricetus auratus**<br>golden hamster	**695**	XM_005070431.4
**Dipodomys ordii**<br>Ord’s kangaroo rat	**713**	XM_013022573.1
**Castor canadensis**<br>American beaver	**701**	XM_020175016.1
**Mus caroli**<br>Ryukyu mouse	**728**	XM_029483590.1
**Mus pahari**<br>shrew mouse	**550**	XM_029545237.1
**Peromyscus maniculatus bairdii**<br>prairie deer mouse	**699**	XM_006974797.2
**Jaculus jaculus**<br>lesser Egyptian jerboa	**921**	XM_004665507.1
**Meriones unguiculatus**<br>Mongolian gerbil	**699**	XM_021626659.1
**Rattus rattus**<br>black rat	**471**	XM_032916924.1
**Grammomys surdaster**<br>thicket rat	**260**	XM_028778791.1
**Mastomys coucha**<br>southern multimammate mouse	**495**	XM_031341979.1
**Arvicanthis niloticus**<br>African grass rat	**641**	XM_034508367.1
**Cricetulus griseus**<br>Chinese hamster	**703**	XM_003502153.5
**Peromyscus leucopus**<br>white-footed mouse	**782**	XM_028876357.2
**Onychomys torridus**<br>southern grasshopper mouse	**696**	XM_036201671.1
**Arvicola amphibius**<br>Eurasian water vole	**704**	XM_038339639.1
**Microtus oregoni**<br>creeping vole	**705**	XM_041669288.1
**Microtus ochrogaster**<br>prairie vole	**706**	XM_026788902.1
**Cavia porcellus**<br>domestic guinea pig	**686**	XM_013150060.2
**Octodon degus**<br>degu	**702**	XM_023720875.1
**Fukomys damarensis**<br>Damaraland mole-rat	**693**	XM_033762286.1
**Marmota marmota**<br>alpine marmot	**551**	XM_015479628.1
**Ictidomys tridecemlineatus**<br>thirteen-lined ground squirrel	**705**	XM_005322047.4
**Urocitellus parryii**	**705**	XM_026387235.1

Arctic ground squirrel		
**Marmota flaviventris**<br>yellow-bellied marmot	**705**	XM_027934553.1

b. Carnivore sequences analyzed in this study.

Organism	Length of amino acid sequence	Accession ID
**Canis lupus familiaris**<br>dog	716	XM_005626140.4
**Mustela putorius furo**<br>domestic ferret	729	XM_004742028.2
**Panthera tigris altaica**<br>Amur tiger	665	XM_007075417.1
**Enhydra lutris kenyoni**<br>northern sea otter	720	XM_022504396.1
**Acinonyx jubatus**<br>cheetah	715	XM_027072376.1
**Lynx canadensis**<br>Canada lynx	715	XM_030310672.1
**Puma yagouaroundi**<br>jaguarundi	715	XM_040463327.1
**Puma concolor**<br>puma	714	XM_025932404.1
**Felis catus**<br>domestic cat	715	XM_003984033.5
**Panthera pardus**<br>leopard	715	XM_019431779.1
**Canis lupus dingo**<br>dingo	716	XM_025468782.2
**Ursus arctos horribilis**<br>grizzly bear	719	XM_026485066.1
**Lontra canadensis**<br>North American river otter	721	XM_032873831.1
**Mustela erminea**<br>ermine	729	XM_032353647.1
**Odobenus rosmarus divergens**<br>Pacific walrus	719	XM_004397721.1
**Hyaena hyaena**<br>striped hyena	705	XM_039240346.1
**Suricata suricatta**<br>meerkat	706	XM_029937442.1
**Vulpes lagopus**<br>Arctic fox		XM_041757534.1
**Vulpes vulpes**<br>red fox	776	XM_025992735.1
**Ursus maritimus**<br>polar bear	719	XM_040623440.1
**Ailuropoda melanoleuca**<br>giant panda	720	XM_034659197.1
**Eumetopias jubatus**<br>Steller sea lion	718	XM_028089669.1
**Zalophus californianus**<br>California sea lion	718	XM_027624320.1
**Neomonachus schauinslandi**<br>Hawaiian monk seal	721	XM_021699187.1
**Halichoerus grypus**<br>grey seal	722	XM_036121192.1

**Leptonychotes weddellii**<br>Weddell seal	722	XM_006733195.2
**Mirounga leonina**<br>southern elephant seal	720	XM_035001465.1
**Phoca vitulina**<br>harbor seal	722	XM_032410896.1

c. Ungulate sequences analyzed in this study.

Organism	Length of amino acid sequence	Accession ID
**Bos taurus**<br>cattle	721	XM_005212825.4
**Odocoileus virginianus texanus**<br>Texas whitetail deer	716	XM_020891631.1
**Bison bison**<br>American bison	721	XM_010863278.1
**Bos indicus x Bos taurus**<br>hybrid cattle	721	XM_027555592.1
**Bos mutus**<br>wild yak	717	XM_005889728.2
**Bos indicus**<br>zebu	710	XM_019969488.1
**Bubalus bubalis**<br>water buffalo	721	XM_006049386.2
**Oryx dammah**<br>scimitar-horned oryx	810	XM_040224006.1
**Capra hircus**<br>goat	722	XM_013967738.2
**Ovis aries**<br>sheep	722	XM_027966713.2
**Sus scrofa**<br>pig	720	XM_003354802.4
**Vicugna pacos**<br>alpaca	723	XM_015237028.2
**Camelus ferus**<br>wild Bactrian camel	723	XM_014564859.2
**Camelus bactrianus**<br>Bactrian camel	552	XM_010951902.1
**Camelus dromedarius**<br>Arabian camel	722	XM_010989773.2
**Neophocaena asiaeorientalis**<br>Yangtze finless porpoise	713	XM_024753025.1
**Balaenoptera acutorostrata scammony**<br>minke whale	717	XM_007189708.1
**Lipotes vexillifer**<br>Yangtze River dolphin	713	XM_007464134.1
**Lagenorhynchus obliquidens**<br>Pacific white-sided dolphin	717	XM_027131791.1
**Globicephala melas**<br>long-finned pilot whale	717	XM_030877335.1
**Orcinus orca**<br>killer whale	717	XM_004280649.2
**Tursiops truncatus**<br>common bottlenose dolphin	717	XM_019943077.2
**Phocoena sinus**	713	XM_032653378.1

vaquita		
**Monodon monoceros**<br>narwhal	717	XM_029232453.1
**Delphinapterus leucas**<br>beluga whale	717	XM_022597296.2
**Physeter catodon**<br>sperm whale	727	XM_024133601.2
**Balaenoptera musculus**<br>blue whale	**727**	XM_036873937.1

d. Primate sequences analyzed in this study.

Organism	Length of amino acid sequence	Accession ID
**Homo sapiens**<br>human	716	NM_001201543.2
**Pan troglodytes**<br>chimpanzee	716	XM_515502.6
**Macaca mulatta**<br>rhesus macaque	716	XM_028831643.1
**Macaca fascicularis**<br>crab-eating macaque	716	XM_005575777.2
**Callithrix jacchus**<br>white-tufted-ear marmoset	715	XM_003734729.4
**Colobus angolensis palliates**<br>Angola black-and-white colobus	562	XM_011941831.1
**Chlorocebus sabaeus**<br>green monkey	716	XM_007970494.2
**Cercocebus atys**<br>sooty mangabey	714	XM_012039548.1
**Macaca nemestrina**<br>pig-tailed macaque	716	XM_011713355.2
**Papio anubis**<br>olive baboon	716	XM_003908708.5
**Theropithecus gelada**<br>gelada	716	XM_025354341.1
**Mandrillus leucophaeus**<br>drill	716	XM_011987312.1
**Trachypithecus francoisi**<br>François' langur	711	XM_033239161.1
**Rhinopithecus bieti**<br>black snub-nosed monkey	711	XM_017889712.1
**Rhinopithecus roxellana**<br>golden snub-nosed monkey	711	XM_010384442.2
**Piliocolobus tephrosceles**<br>Ugandan red colobus	711	XM_023228337.1
**Gorilla gorilla**<br>western gorilla	716	XM_004029293.3
**Pan paniscus**<br>pygmy chimpanzee	716	XM_003830874.3
**Pongo abelii**<br>Sumatran orangutan	716	NM_001374141.1
**Nomascus leucogenys**<br>northern white-cheeked gibbon	716	XM_003262434.4
**Hylobates moloch**<br>silvery gibbon	716	XM_032177370.1
**Saimiri boliviensis**<br>Bolivian squirrel monkey	715	XM_039464018.1

**Sapajus apella**<br>tufted capuchin	715	XM_032292015.1
**Cebus imitator**<br>Panamanian white-faced capuchin	715	XM_017522196.2
**Aotus nancymaae**<br>Ma’s night monkey	715	XM_012477168.2
**Carlito syrichta**<br>Philippine tarsier	715	XM_008054794.2
**Propithecus coquereli**<br>Coquerel’s sifaka	714	XM_012639195.1
**Microcebus murinus**<br>gray mouse lemur	708	XM_012764864.2
**Otolemur garnettii**<br>small-eared galago	712	XM_003787945.1

References

1 Plant, T. M. & Zeleznik, A. J. *Knobil and Neill's physiology of reproduction*. (Academic Press, 2014).

2 Murrell, B. *et al.* Detecting individual sites subject to episodic diversifying selection. *PLoS Genet* **8**, e1002764 (2012). https://doi.org:10.1371/journal.pgen.1002764

3 Kosakovsky Pond, S. L. & Frost, S. D. Not so different after all: a comparison of methods for detecting amino acid sites under selection. *Molecular biology and evolution* **22**, 1208-1222 (2005).

4 Green, C. D. *et al.* A Comprehensive Roadmap of Murine Spermatogenesis Defined by Single-Cell RNA-Seq. *Dev Cell* **46**, 651-667 e610 (2018). https://doi.org:10.1016/j.devcel.2018.07.025
