# Supplementary material for: The evolution of centriole degradation in mouse sperm
Source: Nat Commun. 2024 Jan 2;15:117. doi: 10.1038/s41467-023-44411-8 (PMC10761967; doi:10.1038/s41467-023-44411-8)
Supplement: Supplementary file 3 — Description of Additional Supplementary Files [file 41467_2023_44411_MOESM3_ESM.pdf]

Description of Additional Supplementary Files:

Supplementary Data 1: Survey of original literature on rodent spermatozoan ultrastructure.

Supplementary Data 2: Identity Ratio range of sperm centrosomal proteins.
