# Supplementary material for: The evolution of centriole degradation in mouse sperm
Source: Nat Commun. 2024 Jan 2;15:117. doi: 10.1038/s41467-023-44411-8 (PMC10761967; doi:10.1038/s41467-023-44411-8)
Supplement: Supplementary file 4 — Supplementary Data 1 [file 41467_2023_44411_MOESM4_ESM.pdf]

Supplementary Data 1 Survey of original literature on rodent spermatozoan ultrastructure

Below are sections from selected papers that summarize the literature pertaining to sperm centriole status, followed by Excerpts and figures describing centriole status from additional manuscripts. The information is organized by Suborders, Species, and Papers.

Suborder Hystricomorpha – with proximal centriole

A proximal centriole was reported in all studied rodent species of **suborder Hystricomorpha**, which includes the guinea pig (*Cavia porcellus*) (Fawcett, 1965; Gordon and Bensch, 1968), chinchilla (*Chinchilla lanigera*) (Healey and Weir, 1970), cane rat (*Thryonomys swinderianus*) (Soley, 2016), agouti (*Dasyprocta aguti*) (Arroyo et al., 2017), Spix's yellow-toothed cavy (*Galea spixii*) (Santos et al., 2014), and naked mole rat (*Heterocephalus glaber*) (Van Der Horst et al., 2011).

Guinea pig (*Cavia porcellus*) – Fawcett

Fawcett, D.W. 1965. The anatomy of the mammalian spermatozoon with particular reference to the guinea pig. *Z Zellforsch Mikrosk Anat*. 67:279-296.

Excerpts on centriole status

Page 287: Fig 4 – "A **centriole** (*Ce*) is lodged in the interior of the connecting piece."

Page 289: "The **centriole** is situated in the interior of the connecting-piece immediately beneath its articular surface, and is set at an angle of about 45° to the axis of the tail with one end opposed to the oblique inner surface of the capitellum and the other end occupying a niche in the inner aspect of the other major column (Figs. 3B and 4)."

Figures on centriole status

Fig 3B

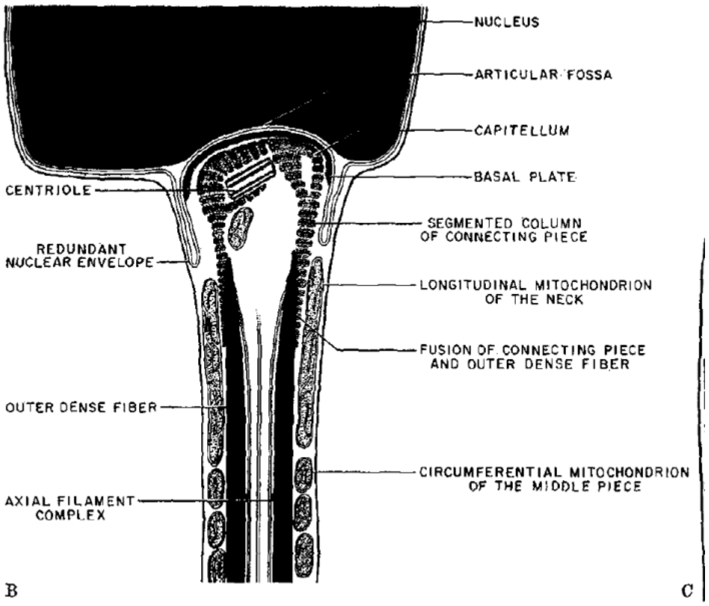

Fig 4

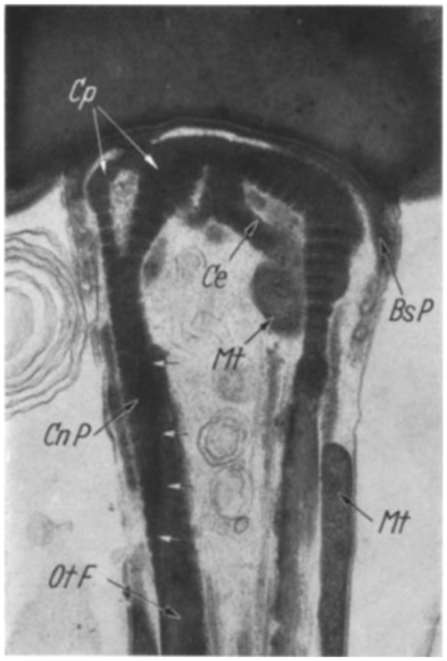

Permission to copy and redistribute the figure is by License of original publication: Owned by Springer Nature; RightsLink order number 501857384.

Guinea pig (*Cavia porcellus*) – Gordon

Gordon, M., and K.G. Bensch. 1968. Cytochemical differentiation of the guinea pig sperm flagellum with phosphotungstic acid. *J Ultrastruct Res*. 24:33-50.

Excerpts on centriole status

Page 44: "The outlines of the **proximal centriole**, of which only the medial portion is visible, are stained with E-PTA (Fig. 18)."

Page 42: Fig. 18 **–** "The neck of the spermatozoon shows PTA-dense material in the crevices of the segmented columns (short arrow). The walls of the **proximal centriole** (*c*) have deposits of PTA-stained substance. The long arrow points to gap in the contact area between the coarse fiber (*f*) and the segmented column. × 41,000."

Figures on centriole status

Fig 18

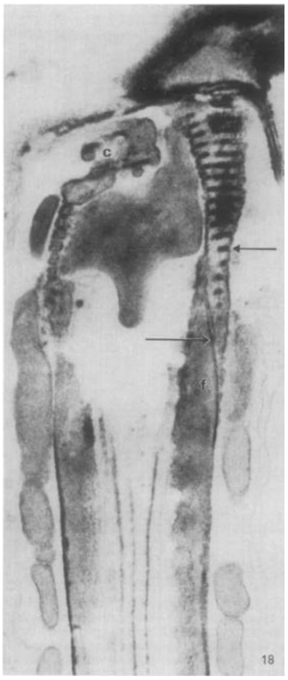

Permission to copy and redistribute the figure is by License of original publication; RightsLink order number 501857384.

Chinchilla (*Chinchilla lanigera*) – Healey

Healey, P., and B.J. Weir. 1970. Changes in the ultrastructure of chinchilla spermatozoa in different diluents. *J Reprod Fertil*. 21:191-193.

Excerpts on centriole status

Page 192: “Two electron-dense fibres terminate at the single neck **centriole** (Pl. 1, Fig. 2)”

Page 192: Explanation of Plate 1 – “Figs. 1 to 3 are electron micrographs of chinchilla spermatozoa fixed directly in osmium tetroxide and stained with lead citrate. AC = acrosome; AT = acrosome termination; C = **centriole**;… Fig. 2. Sagittal section of neck region, ×40,000.”

Figures on centriole status

Pl. 1, Fig. 2

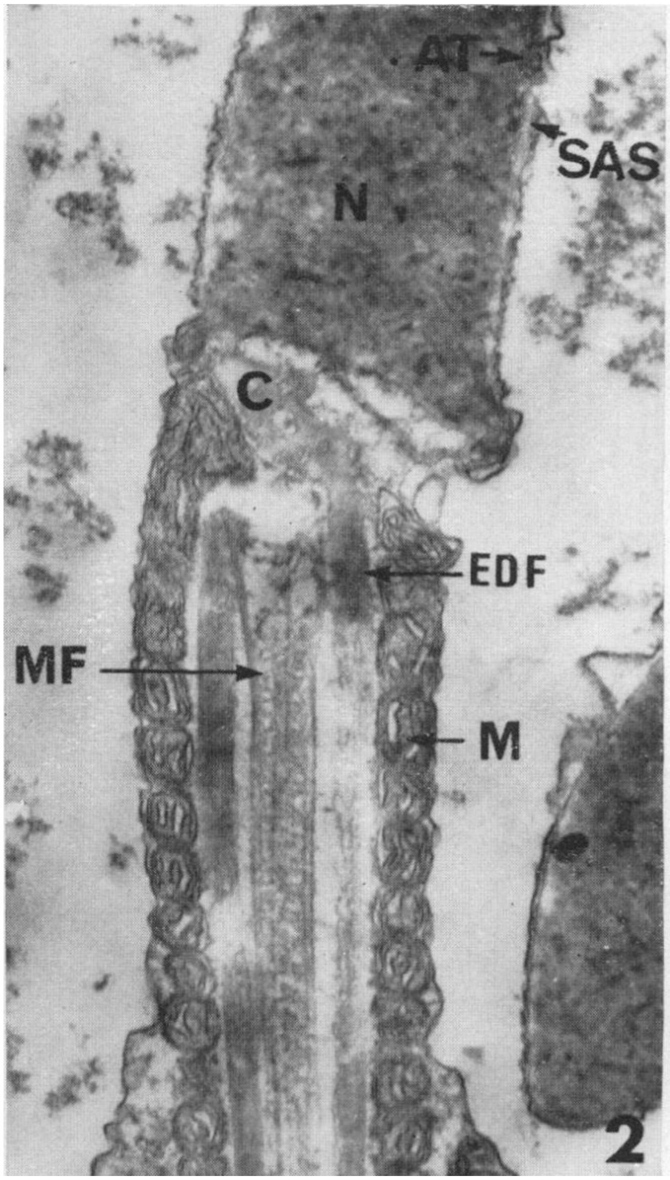

Permission to copy and redistribute the figure is by CCC Marketplace Request ID 600142457.

Cane rat (*Thryonomys swinderianus*) – Soley

Soley, J.T. 2016. A comparative overview of the sperm centriolar complex in mammals and birds: Variations on a theme. *Animal reproduction science*. 169:14-23.

Excerpts on centriole status

Page 15: "The orientation of the **centrioles** may also differ. In most species the paired **centrioles** are typically aligned at right angles to each other, as also observed in the cane rat and cheetah (Fig. 1)."

Page 16: Fig 1b legend - "(b) Neck region of an ejaculated cane rat sperm showing the **proximal** (white star) and **distal** (black star) **centrioles**, dense fibers (chevrons), redundant nuclear membrane (block arrows) and striated columns (Sc). Note the empty/disrupted appearance of the **distal centriole**."

Figures on centriole status

Fig 1b

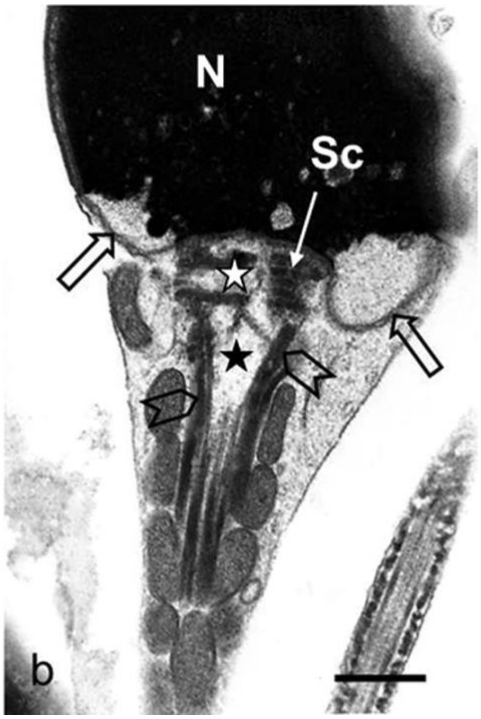

Permission to copy and redistribute the figure is by License of original publication: RightsLink order number 501857392.

Agouti (*Dasyprocta aguti*) – Arroyo

Arroyo, M.A.M., F.F.S. Silva, P.R.S. Santos, A.R. Silva, M.F. Oliveira, and A.C.A. Neto. 2017. Ultrastructure of spermatogenesis and spermatozoa in agoutis during sexual development. *Reprod Fertil Dev*. 29:383-393.

Excerpts on centriole status

Page E: "With regard to the connecting piece ('neck'), it communicated with the head through the implantation fossa and capitulum. From this point it was possible to see the **proximal centriole** (Fig. 7*c*). However, a possible degeneration of the **proximal** and **distal centrioles**, characterised by centriolar space, was noted."

Page H: "Fig. 7. Ultrastructure of spermatozoon of agouti (*Dasyprocta aguti*) visualised by transmission electron microscopy. (*a*–*b*) Tapered head. (*c*–*d*) Connecting piece ('neck'). a, acrosome; AF, axial filaments; am, acrosomal membrane; ax, acrosomal matrix; ca, capitulum; **ce, proximal centriole**; cs, centriolar space; cye, cytoplasmic extensions; DF, dense fibres; if, implantation fossa; Mi, mitochondria; N, nucleus; pt, perinuclear theca; ♦ evidence of the **distal centriole**."

Figures on centriole status

Fig 7c and 7d (proximal centriole is detected in both)

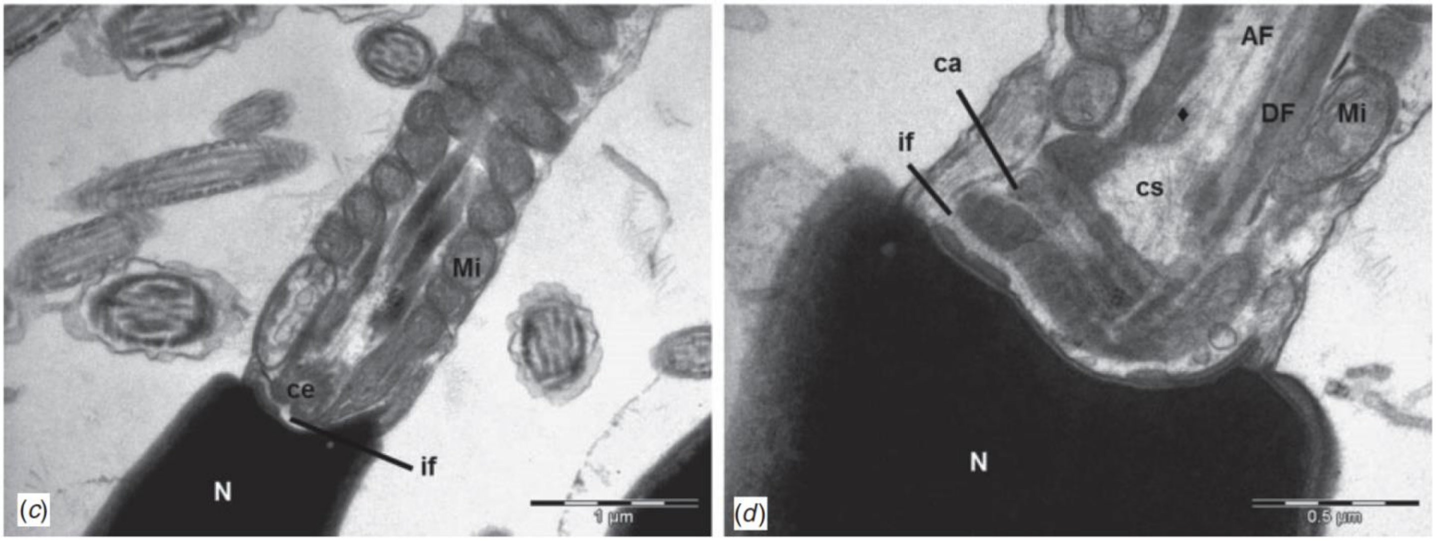

PAID License of original publication - Order number: 1413770

Santos, P.R., M.F. Oliveira, M.A. Arroyo, A.R. Silva, R.E. Rici, M.A. Miglino, and A.C. Assis Neto. 2014. Ultrastructure of spermatogenesis in Spix's yellow-toothed cavy (Galea spixii). *Reproduction*. 147:13-19.

Excerpts on centriole status

Page 17: "Figure 6 Ultrastructure of spermatozoa, Spix's yellow-toothed cavy (SYC, *Galea spixii*). Longitudinal section of the spermatozoon (a, acrosome; Af, axial filament; An, ring; Bp, basal plate; Es, equatorial segment; Fs, fibrous sheath; m, mitochondria; N, nucleus; Od, outer dense fibers; P, perforatorium; **Pc, proximal centriole**; Pm, plasma membrane). Transmission electron microscopy; scale bars: 2 µm."

Page 17: "Extending from the inner acrosomal membrane into the middle of the acrosome is the perforatorium. The core was chromatin dense and homogeneous in shape (Fig. 6). The neck was located in the hollow core of the base where the **proximal centriole** attached to the basal plate and the nuclear membrane. The middle piece was shorter and contained mitochondrial pairs. The diameter decreased in the tail."

Figures on centriole status

Figure 6

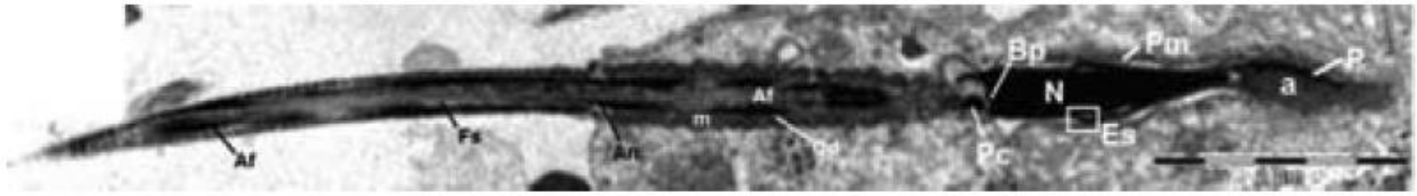

Permission to copy and redistribute the figure is by CCC Marketplace Request ID 600142458

Naked mole rat (*Heterocephalus glaber*) – Van Der Horst

Van Der Horst, G., L. Maree, S.H. Kotze, and M.J. O'Riain. 2011. Sperm structure and motility in the eusocial naked mole-rat, Heterocephalus glaber: a case of degenerative orthogenesis in the absence of sperm competition? *BMC Evol Biol*. 11:351.

Excerpts on centriole status

Page 3: "Figure 2 Scanning and transmission electron micrographs of naked mole-rat spermatozoa. a) Scanning electron micrograph of a typical multi-lobed sperm head, b-h) Transmission electron micrographs of naked mole-rat spermatozoa sectioned in different planes: b) Longitudinal section almost similar to spermatozoon in (a) showing irregular arranged chromatin, small midpiece and thin tail; c) Two sperm heads showing severe fragmentation and small midpiece; d) Sperm head containing a simple acrosomal cap; d-e) **Proximal centriole** with capitulum and striations of cross banded structures; f) Transverse to oblique section of anterior part of midpiece showing mitochondria with tubular-like cristae, some cross banded structures and **distal centriole**; g) Axoneme (9+2 microtubules) surrounded by nine outer dense fibres in midpiece; h) Only axonome in tail principal piece without a fibrous sheath. ACR = acrosome, HEAD = sperm head, FRG = fragmented sperm head, CAP = capitulum, **PC = proximal centriole**, CBS = cross banded structures, **DC = distal centriole**, MIT = mitochondria, MP = midpiece. Scale bars: a, b, c = 1 μm; d, e, f = 0.5 μm; g, h = 0.25 μm."

Page 4: "These nine cross banded structures furthermore connect with the outer nine fibres close to the **distal centriole**. Just below the capitulum and surrounded by the cross banded structures is a clearly demarcated **proximal centriole** (Figures 2d and 2e) which is 90° orientated in terms of its central axis to the **distal centriole**. The **distal centriole** (Figure 2f) gives rise to the axoneme, which typically has the 9+2 microtubule arrangement."

Figures on centriole status

Fig 2d–2f

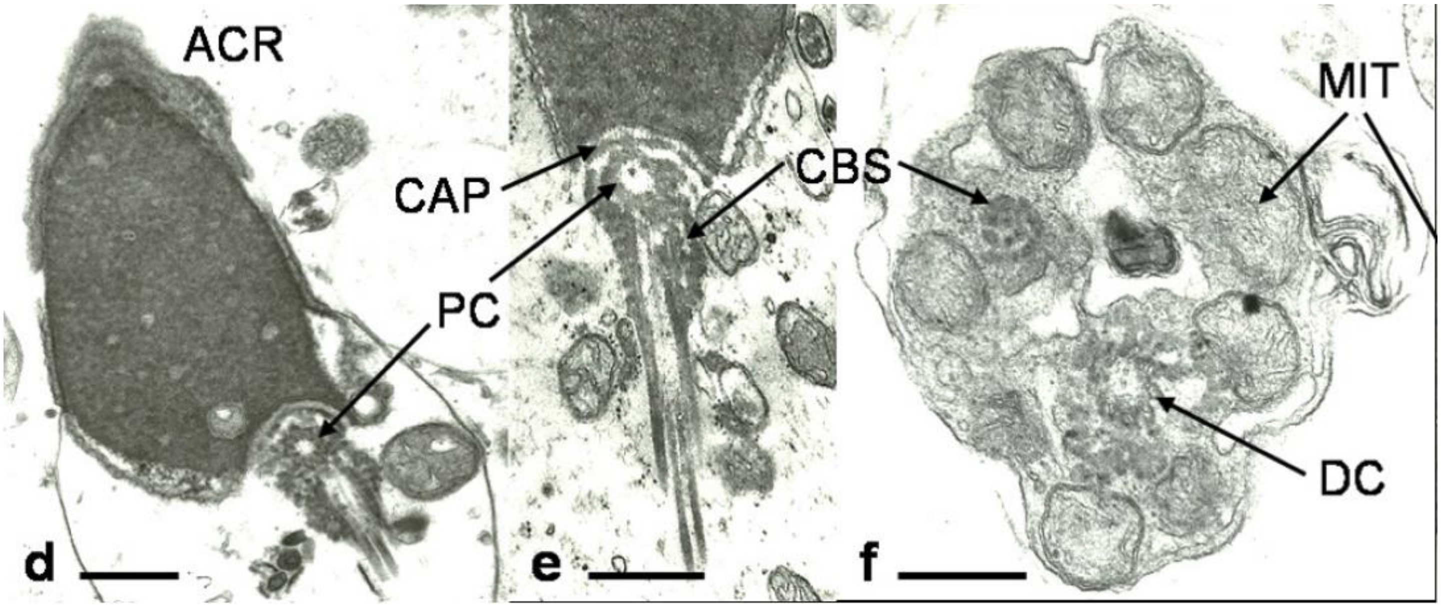

Permission to copy and redistribute the material is by CC BY 2.0 DEED license (https://creativecommons.org/licenses/by/2.0/deed.en) and was also obtained from Dr. Liana Maree, the corresponding author.

Suborder Sciuromorpha – with proximal centriole

A proximal centriole is also reported in the rodent species of **suborder Sciuromorpha**, which includes the Korean squirrel (*Tamias sibiricus*) (Lee and Park, 2011) and the flying squirrel (*Glaucomys volans*) (Hruban et al., 1971).

Lee, J.-H., and K.-R. Park. 2011. Fine Structure of Sperm in the Korea Squirrel, Tamias sibiricus. Applied Microscopy. 41:99-107.

Excerpts on centriole status

Page 100: "정자 경부는 핵의 아래면과 접하고 있는 기저판 (Basal plate, Bp) 을 비롯하여 (Fig. 2a, b), 기저판 아래면의 근위 중 심체 (Proximal centriole, Pc) 와 원위 중심체 (Distal centriole, Dc) 그리고 이들 중심체들의 외곽에는 9개의 분절주 (Segmented columns, Sc) 가 존재하고 있었다 (Figs. 2a, b, 4(a-c))." *Google translation*: "The sperm neck includes the basal plate (Bp), which is in contact with the lower surface of the nucleus (Fig. 2a, b), and the **proximal centriole (Pc**) and **distal centriole (Dc)** on the lower surface of the basal plate. And there were 9 segmented columns (Sc) on the outside of these centrosomes (Figs. 2a, b, 4(a-c))."

Page 104: "Fig. 2. Sagittal sections of sperm head, neck, middle piece and principal piece in *T. sibiricus*. (2a) The mitochondria are arranged at the sides of axoneme regularly, and total number of the mitochondrial gyres were 26. (2b) Cross sections in 2b in approximate regions a-a′, b-b′, c-c′ and d-d′ shown in 4a, 4b, 4c and 4d, respectively. The segmented columns (Sc) were surrounded by redundant membranous scroll (arrows). The segmented columns was about 10 ~ 12 in number. Note the electron dense materials (asterisks) are observed in the neck region. An, annulus; Cd, cytoplasmic droplet; **Dc, distal centriole**; H, head; Mp, middle piece; Nc, neck; M, mitochondria; N, nucleus; Odf, outer dense fiber; **Pc, proximal centriole**; Pm, plasma membrane; Pp, principal piece; Sc, segmented columns."

Flying squirrel (*Glaucomys volans*) – Hruban

Hruban, Z., J. Martan, and I. Aschenbrenner. 1971. Polarized cylindrical body in the epididymis of the flying squirrel. *Journal of Morphology*. 135:87-97.

Excerpts on centriole status

Page 88: “These structures are the connecting piece attached to the basal plate anteriorly and to the outer dense fibers posteriorly, one or two mitochondria, a **centriole** and the redundant portion of the nuclear envelope forming a single fold (fig. 9).”

Figures on centriole status

Comment: Fig 9 does not include the centriole.

Page 92: “9) Portion of the neck of a spermatozoon. Redundant nuclear envelope is seen at arrowhead. Portion of a nucleus (N), basal plate (arrow) and segmented column (C) of the connecting piece are seen on the right. Portion of the cytoplasmic droplet is seen on the left. × 27,000.”

Suborder Myomorpha – with proximal centriole

Finally, a proximal centriole is reported in three studied species of family Cricetidae of **suborder Myomorpha**: golden hamster (*Mesocricetus auratus*) (Franklin et al., 1970; Hamasaki et al., 1994), Chinese hamster (*Cricetulus griseus*) (Yanagimachi et al., 1983), and Winkelmann's mouse (*Peromyscus winkelmanni*) (Garcia Lorenzana et al., 1998). The presence of a proximal centriole in the three main rodent suborders suggests that a canonical proximal centriole was present in the last common rodent ancestor and is present in many extant rodent species.

Golden hamster (*Mesocricetus auratus*) – Franklin

Franklin, L.E., C. Barros, and E.N. Fussell. 1970. The acrosomal region and the acrosome reaction in sperm of the golden hamster. *Biology of reproduction*. 3:180-200.

Excerpts on centriole status

Page 181: "The two flattened surfaces of the mammalian sperm head are customarily designated as the dorsal and ventral surfaces. According to that designation the long axis of the **proximal centriole** lies approximately in a horizontal plane (Figs. 1 and 2), and in golden hamsters the prominent acrosomal region curves strongly to one side (Figs. 1–4)."

Page 182: "FIG. 1. Diagram of the head of the golden hamster spermatozoon as it appears in sections cut in several different planes. The central figure represents the midhorizontal plane and serves as a reference for the construction of other sections. Section A-A' at the left is parasagittal section through the curved acrosomal region. Examples of parasagittal sections are Fig. 5–7. Section B-B' above the plan view is a transverse section across the acrosomal region. Examples are Fig. 9 and 10. Section C-C' at the right is a midsagittal section. Figure 8 includes a similar section near the midsagittal plane. The perforatorium (stippled area) continues from the curved acrosomal region back around the front half of the nucleus as the subacrosomal space and its contents."

Page 182: "FIG. 2. Horizontal section of a relatively late spermatid. The longitudinal axis of the **proximal centriole** (arrow) lies in a horizontal plane. The **proximal centriole** is more clearly seen in the stage of spermiogenesis shown in the figure than in mature sperm, because the distal portion of the **proximal centriole** subsequently disappears (Fawcett and Phillips, 1967)"

Figures on centriole status

Fig 1

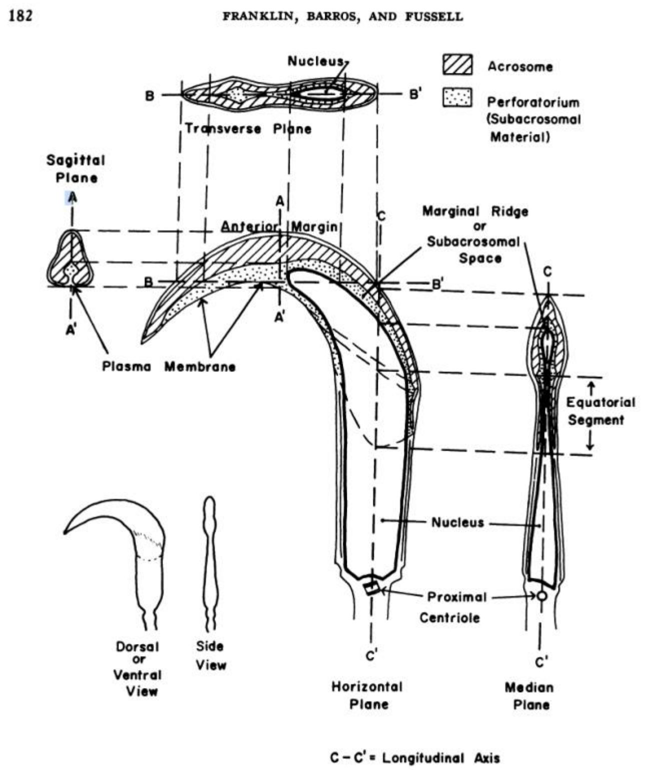

Fig 2

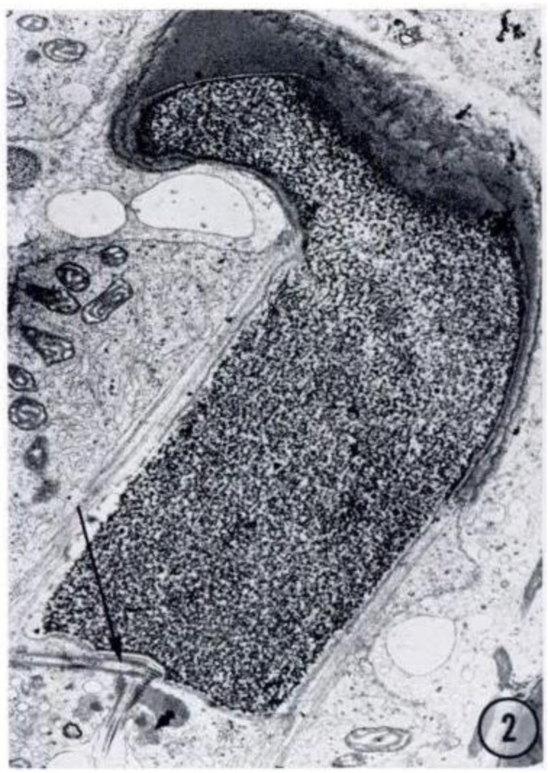

License of original publication was paid - order number 5663131142113

Golden hamster (*Mesocricetus auratus*) – Hamasaki

Hamasaki, M., M. Wakimoto, T. Maehara, and H. Matsuo. 1994. Three-dimensional structures of the neck region of the hamster spermatozoa in the caudal epididymis. *Arch Histol Cytol*. 57:59-65.

Excerpts on centriole status

Page 60: "Fig. 2. Higher magnification of the neck region of a mature spermatozoon. Basal plate (*BP*), capitulum (*C*), **proximal centriole** (***PC***), striated column (*S*) and abundant nuclear membrane (*). ×43,000"

Page 61: "Fig. 3. The horizontal plane of the neck region of a mature spermatozoon. The first right-side mitochondrium protrudes innerward to form a triangular pyramid. Large (*L*) and small (*S*) plates of the capitulum; **proximal centriole (*PC*)** and abundant nuclear membrane (*). ×57,000"

Page 61: "The center of the neck region was composed of the basal plate, the capitulum, the **proximal centriole** and the space which the **distal centriole** had occupied inside the striated column, the first mitochondria, and the redundant nuclear envelope... Figure 3 shows a horizontal section of the neck portion of a spermatozoon. Some edges of the right mitochondrium protrude inward to push the caudal part of the **proximal centriole** toward the left side, and further protrude upward to lift the central area of the capitulum to some extent."

Fig 1

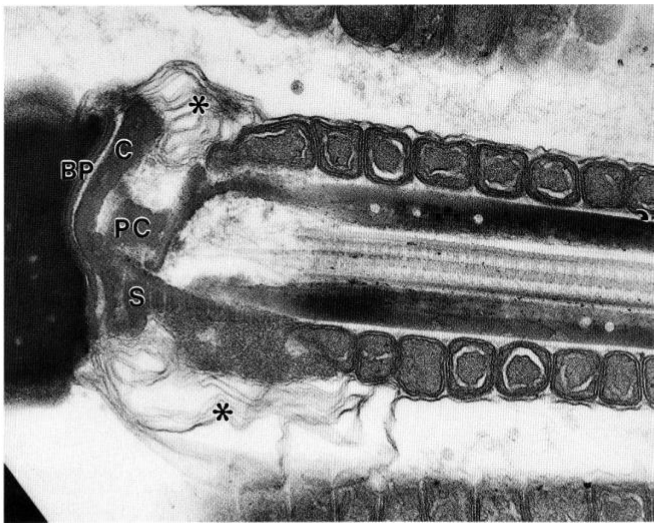

Fig 3

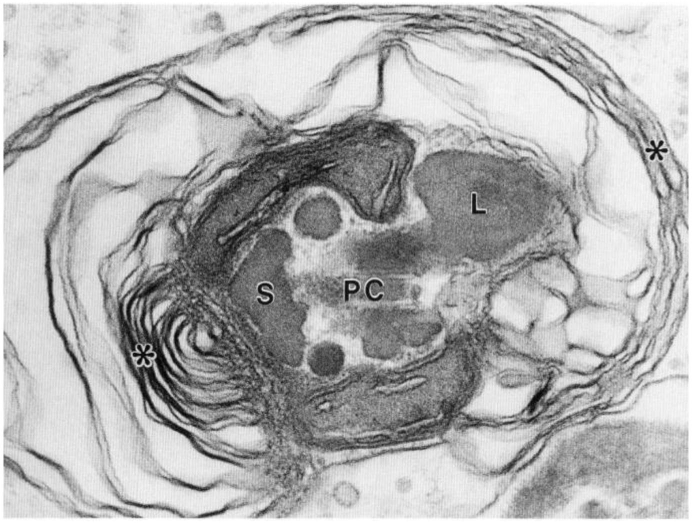

Permisstion was obtained from The Editorial Board of the Archives of Histology and Cytology.

Chinese hamster (*Cricetulus griseus*) – Yanagimachi

Yanagimachi, R., Y. Kamiguchi, S. Sugawara, and K. Mikamo. 1983. Gametes and fertilization in the Chinese hamster. *Gamete research*. 8:97-117.

Quotation on centriole status

Page 114: "Although some sperm tail components (eg, the **proximal centriole**) may be integrated into the vitellus, incorporation of all the sperm tail components in obviously not essential for embryonic development of the zygote at least in the Chinese hamster and field vole."

Figures on centriole status

No image

Winkelmann's mouse (*Peromyscus winkelmanni*) – Garcia Lorenzana

Garcia Lorenzana, M., R. Lopez Wilchis, and G. Vazquez Nin. 1998. Basic aspects of the fine structure of Peromyscus winkelmanni spermatozoa (Rodentia: Cricetidae). *Biblioteca*. 4.

Excerpts on centriole status

Page 93: “The *implantation fossa* appears as a concavity at the base of the head, showing the **centriole** near the fibrous material of the middle piece. (Figure 1)”

Page 94: “Figure 1. A sagittal section of a sperm head of *Peromyscus winkelmanni*. Cz. Clear zones; A. Acrosome; Ss. Subacrosome space; If. Implantation fossa; **Ce. Centriole**. (Magnification 38,000X Bar = 0.5 µm)”

Suborder Myomorpha – without proximal centriole

In contrast, a proximal centriole is reported to be absent in the five studied members of family Muridae of **suborder Myomorpha**, which includes the house mouse (*Mus musculus*) (Leung et al., 2021; Manandhar et al., 1998), rat (*Rattus norvegicus*) (Woolley and Fawcett, 1973), Mongolian gerbil (*Meriones unguiculatus*), and two *Apodemus* species (*Apodemus agrarius coreae* and *Apodemus speciosus peninsulae*) (Chakraborty, 1979; Lee and Mori, 2006). The location of the missing proximal centriole in the spermatozoan neck of these species is marked by an empty, vault-like space, suggesting that a proximal centriole remnant may be present. Indeed, a recent study using state-of-the-art cryo-electron microscopy in house mouse spermatozoa confirmed that there were some microtubule remnants where the proximal centriole should be located (Leung et al., 2021). The absence of a proximal centriole in multiple genera of family Muridae contrasted with its presence in multiple genera of family Cricetidae suggests that proximal centriole degeneration occurred after the divergence of Muridae from Cricetidae, which happened about 20 million years ago (Steppan and Schenk, 2017).

House mouse (*Mus musculus*) – Manandhar

Manandhar, G., P. Sutovsky, H.C. Joshi, T. Stearns, and G. Schatten. 1998. Centrosome reduction during mouse spermiogenesis. *Dev Biol*. 203:424-434.

Excerpts on centriole status

Page 430: "**Centrioles** Degenerate in the Mature Sperm. The **distal centrioles** disintegrate during testicular stage, and by the time the sperm reach epididymis, they are completely lost (Fig. 9)."

Page 431: "FIG. 9. Degeneration of **centrioles** in the epididymal sperm. (A) Section passing longitudinally through the distal and the proximal centriolar regions. The **distal centriole** has degenerated and the space is surrounded by the outer dense fibers (arrows). Few remnants of the centriolar microtubules are visible in the proximal centriolar vault while the majority of them has disappeared. The upper margin of the centriolar vault has folded back into the lumen. (B) Section passing transversely through the **proximal centriolar vault** and obliquely through the distal centriolar region. The extended central microtubule doublet of the axoneme (black arrows) is visible in the distal centriolar vault. **The proximal centriole is in the final stage of degeneration**. Few remnants of microtubules (white arrows) are visible in the finger-like projections, which are the remnants of the spaces previously occupied by the microtubular triplets. (C) Section passing longitudinally through the **proximal centriolar vault** of a mature epididymal sperm. The **proximal centriole** has been lost completely and its vault has collapsed (arrow). Bar, 0.5 µm."

Figures on centriole status

FIG. 9. Degeneration of centrioles in the epididymal sperm.

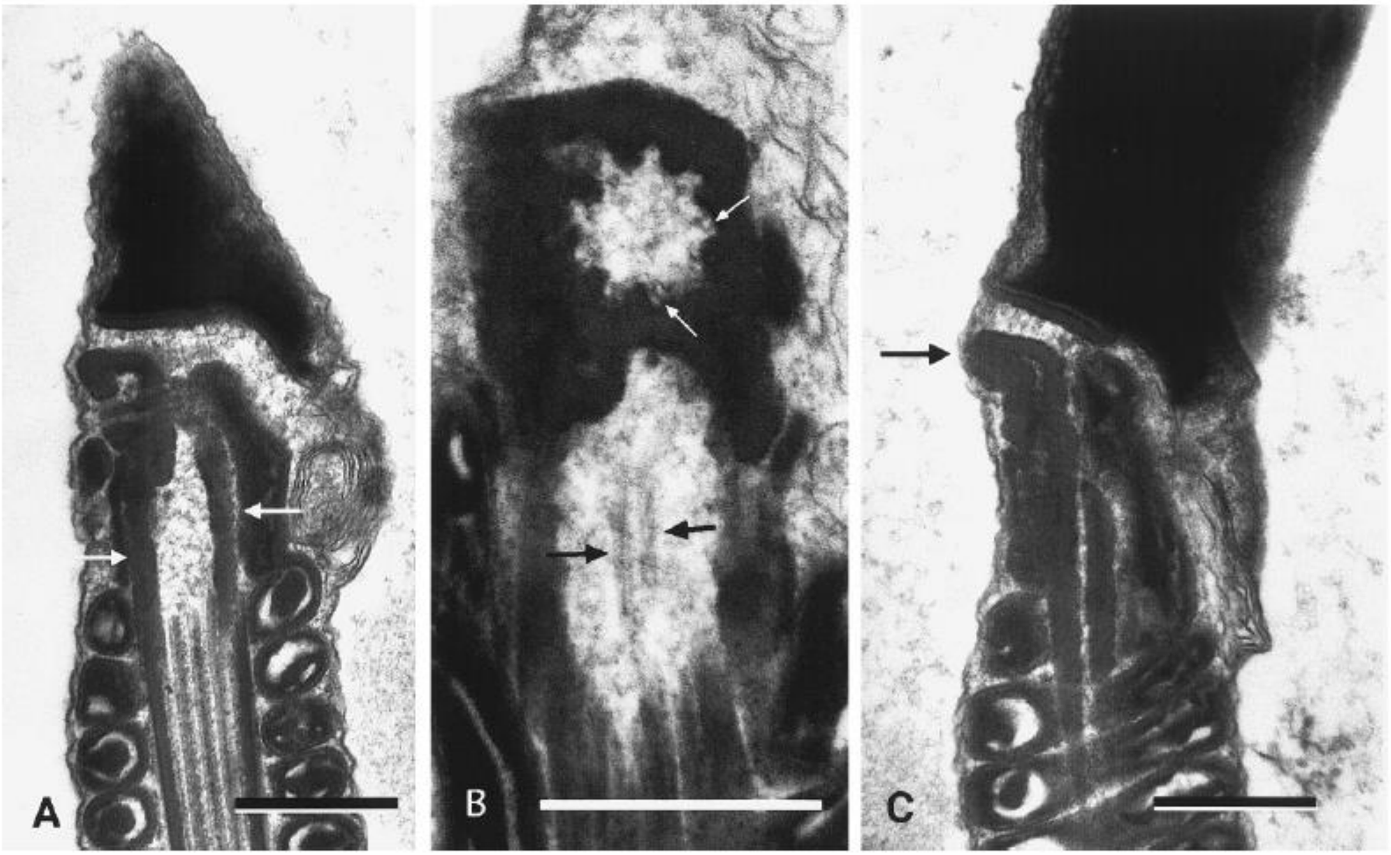

Permission to copy and redistribute the material is by RightsLink order number 501857395.

House mouse (*Mus musculus*) – Leung

Leung, M.R., M.C. Roelofs, R.T. Ravi, P. Maitan, H. Henning, M. Zhang, E.G. Bromfield, S.C. Howes, B.M. Gadella, H. Bloomfield-Gadelha, and T. Zeev-Ben-Mordehai. 2021. The multi-scale architecture of mammalian sperm flagella and implications for ciliary motility. *EMBO J*. 40:e107410.

Excerpts on centriole status

Page 2–3: "Consistent with previous reports that the **PC degenerates** in rodents (Woolley & Fawcett, 1973; Manandhar *et al*, 1998), the **PC was not prominent** in mouse sperm. However, cryo-ET showed unequivocally that some centriolar microtubules remain (Fig 1F), demonstrating that degeneration is incomplete. We observed complete triplets as well as triplets in various stages of degeneration, including triplets in which only the B-tubule had degraded (Fig 1F')."

Page 2: Figure 1 legend – "D–F Tomographic slices through cryo-FIB-milled lamellae of pig (D), horse (E), and mouse (F) sperm. Transverse slices (D'–F') show complete triplets in the pig (D') and the horse (E'), but not in the mouse (F'). **Complete triplets** are indicated by green arrowheads with black outlines, while degenerated triplets are indicated by white arrowheads with green outlines."

Figures on centriole status

Fig 1F and 1F'

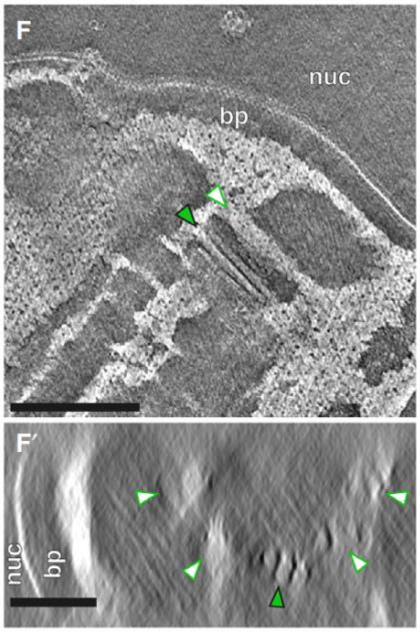

Permission to copy and redistribute the figure is by CC BY-NC-ND 3.0 license (https://creativecommons.org/licenses/by-nc-nd/3.0/deed.en) and was also obtained from Dr. Z. (Tzviya) Zeev Ben Mordehai, the corresponding author.

Rat (*Rattus norvegicus*) – Woolley

Woolley, D.M., and D.W. Fawcett. 1973. The degeneration and disappearance of the centrioles during the development of the rat spermatozoon. *Anat Rec*. 177:289-301.

Excerpts on centriole status

Page 289: “An intensive search has failed to locate a **proximal centriole** in the neck of the rat spermatozoon. This centriole is present in late spermatids but disappears before spermiation. The **distal centriole** also degenerates during spermiogenesis, though more gradually; it is no longer demonstrable by the time the spermatozoa reach the cauda epididymidis (except as a few remnants in a small minority of the cells). The rat spermatozoon is thus exceptional among mammals in being effectively **acentriolate**.”

Page 296: PLATE 1 Legend – “3–4 Transverse sections through mature spermatozoa at a level of the neck corresponding to that in figure 2. The **proximal centriole** (and the adjunct) are absent. A common variation is that a vesicle is present in the vault which formerly contained the **proximal centriole** (fig. 4). × 58,000.”

Figures on centriole status

Fig 3–4

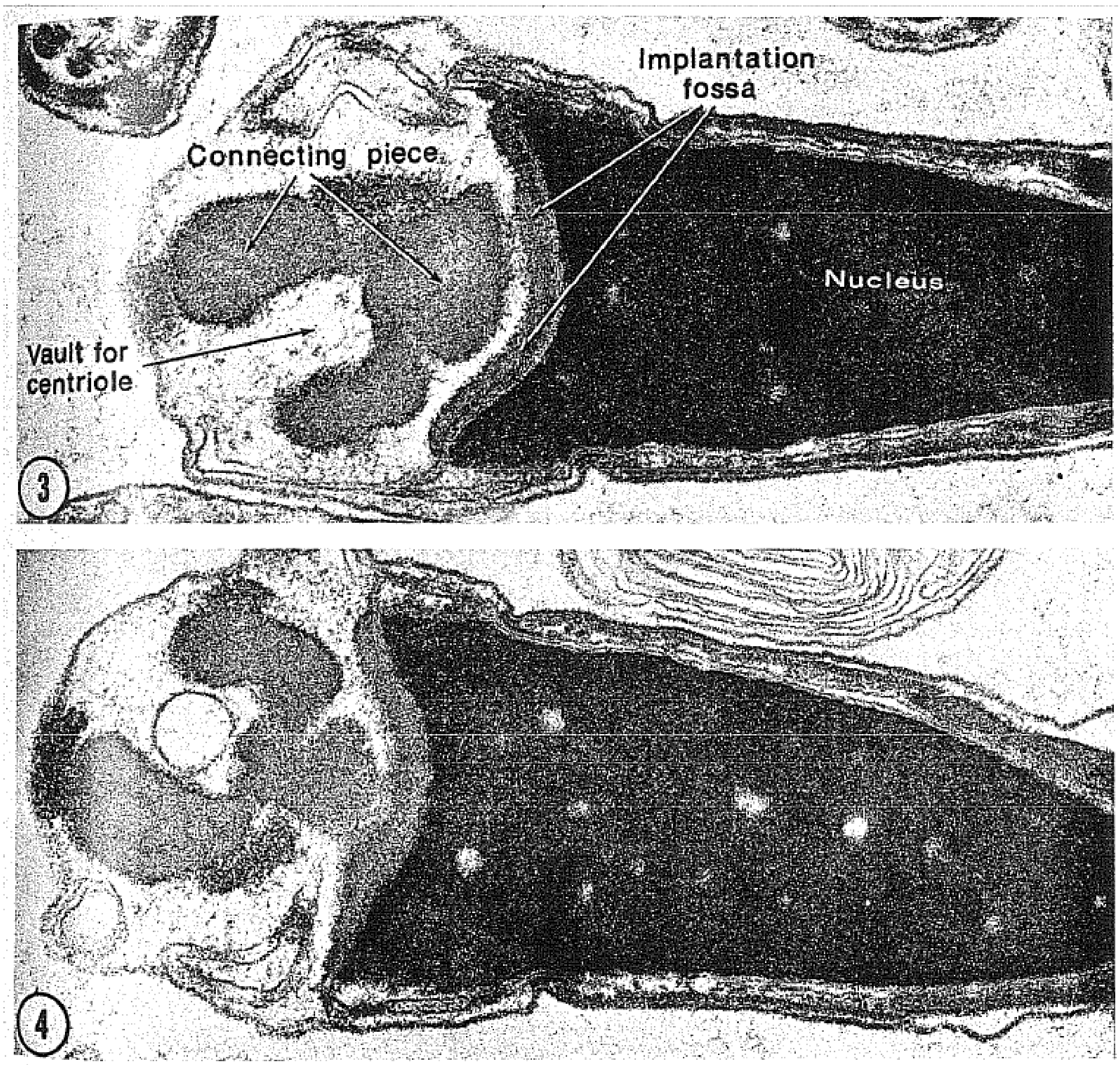

Permission to copy and redistribute the material is by John Wiley & Sons – Books; Request ID: 600142474.

Mongolian gerbil (*Meriones unguiculatus*) – Chakraborty

Chakraborty, J. 1979. Neck region of gerbil spermatozoa. *Gamete Research*. 2:25-34.

Excerpts on centriole status

Page 25: "In the course of the reorganization and degeneration of the **proximal centriole** in the mature acentriolate spermatozoon of the Mongolian gerbil, both the **proximal** and **distal centrioles** appear in the early cap phase of spermatid development."

Page 27: Fig.1 Legend – "H) With further development **remnants of all microtubular components disappear**. Two distinct rudimentary columns (RC) in close association with the mitochondria can be seen at this stage. I) Fully developed neck region of a mature spermatozoa. Three horizontal bars (B) occupy the space of the former **proximal centriole**. Membranous components (M) and a central microtubule occupy the area in which the **distal centriole** was previously located. The rudimentary columns (RC) do not fuse with the capitulum or with other columns."

Page 31: "In mature sperm from the vas deferens, no microtubules were detected in the region of the **proximal centriole** (Figs. 1H and 9)."

Figures on centriole status

Fig 1

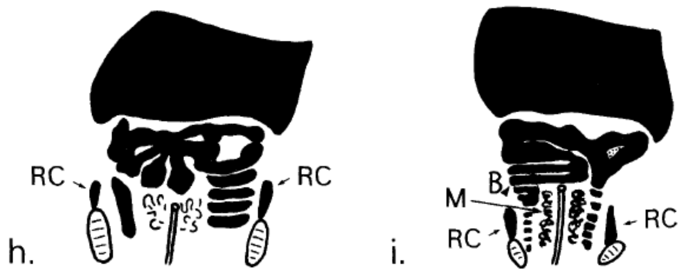

Fig 9

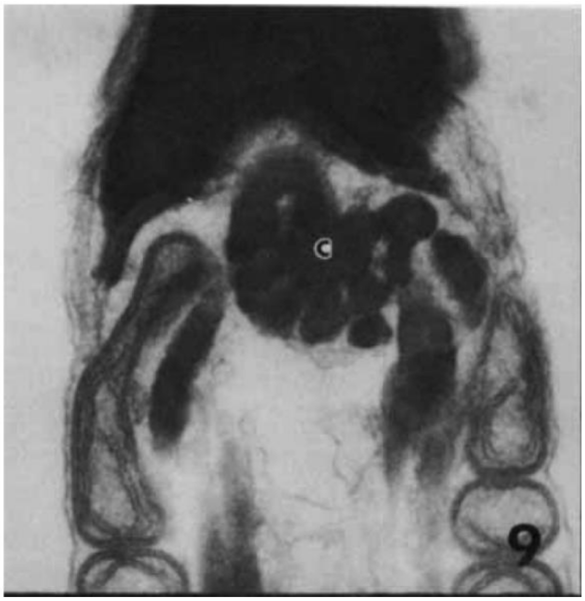

Permission to copy and redistribute the material is by CCC Marketplace Request ID 600142487

Lee, J.-H., and T. Mori. 2006. Ultrastructural observations on the sperm of two Apodemus species, Apodemus agrarius coreae and Apodemus speciosus peninsulae, in Korea.

Excerpts on centriole status

Page 125: "In the neck region, the vault formerly occupied by the **proximal centriole** was retained in a niche in the dense substance of the connecting piece in the spermatozoa of two *Apodemus* species, the mouse and the rat."

Page 128: "Fig. 6. Horizontal section of neck of *A. agrarius coreae*. The **proximal centriole (Pc) was degenerated**. Two ventral spur (Vs) and post-nuclear cap (Pnc) are clearly visible. The mitochondria were presented between segmented column and redundant membrous scoll (Ms). Bp, basal plate; M, mitochondria; Pm, plasma membrane; Se, segmented column. ▲, membranous scroll. Scale bar = 0.5 μm."

Page 129: "Fig. 10. Horizontal section of the neck of *A. speciosus peninsulae*. The **proximal centriole (Pc) was degenerated**. The basal plate (Bp) was adherent to the nuclear envelope, defining the implantation fossa. The segmented columns (Sc) are surrounded by redundant membrous scroll (arrowheads). Developed mitochondria were seen in the neck region. M, mitochondria; Pm, plasma membrane. Scale bar=0.5 µm."

Figures on centriole status

Fig 6

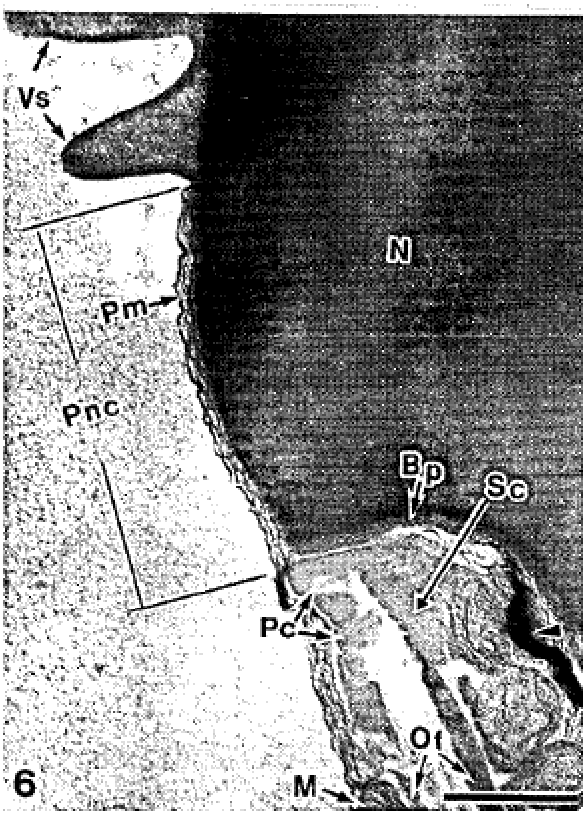

Fig 10

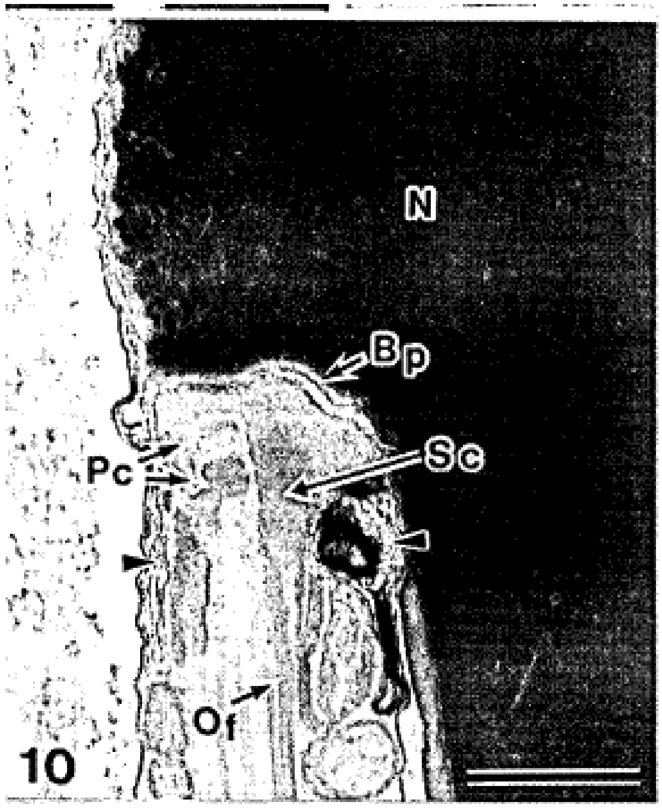

The publisher permitted us to republish.

References

Arroyo, M.A.M., F.F.S. Silva, P.R.S. Santos, A.R. Silva, M.F. Oliveira, and A.C.A. Neto. 2017. Ultrastructure of spermatogenesis and spermatozoa in agoutis during sexual development. *Reprod Fertil Dev*. 29:383-393.

Chakraborty, J. 1979. Neck region of gerbil spermatozoa. *Gamete Research*. 2:25-34.

Fawcett, D.W. 1965. The anatomy of the mammalian spermatozoon with particular reference to the guinea pig. *Z Zellforsch Mikrosk Anat*. 67:279-296.

Franklin, L.E., C. Barros, and E.N. Fussell. 1970. The acrosomal region and the acrosome reaction in sperm of the golden hamster. *Biology of reproduction*. 3:180-200.

Garcia Lorenzana, M., R. Lopez Wilchis, and G. Vazquez Nin. 1998. Basic aspects of the fine structure of Peromyscus winkelmamzi spermatozoa (Rodentia: Cricetidae). *Biblioteca*. 4.

Gordon, M., and K.G. Bensch. 1968. Cytochemical differentiation of the guinea pig sperm flagellum with phosphotungstic acid. *J Ultrastruct Res*. 24:33-50.

Hamasaki, M., M. Wakimoto, T. Maehara, and H. Matsuo. 1994. Three-dimensional structures of the neck region of the hamster spermatozoa in the caudal epididymis. *Arch Histol Cytol*. 57:59-65.

Healey, P., and B.J. Weir. 1970. Changes in the ultrastructure of chinchilla spermatozoa in different diluents. *J Reprod Fertil*. 21:191-193.

Hruban, Z., J. Martan, and I. Aschenbrenner. 1971. Polarized cylindrical body in the epididymis of the flying squirrel. *Journal of Morphology*. 135:87-97.

Lee, J.-H., and T. Mori. 2006. Ultrastructural observations on the sperm of two Apodemus species, Apodemus agrarius coreae and Apodemus speciosus peninsulae, in Korea.

Lee, J.-H., and K.-R. Park. 2011. Fine Structure of Sperm in the Korea Squirrel, Tamias sibiricus. *Applied Microscopy*. 41:99-107.

Leung, M.R., M.C. Roelofs, R.T. Ravi, P. Maitan, H. Henning, M. Zhang, E.G. Bromfield, S.C. Howes, B.M. Gadella, H. Bloomfield-Gadelha, and T. Zeev-Ben-Mordehai. 2021. The multi-scale architecture of mammalian sperm flagella and implications for ciliary motility. *EMBO J*. 40:e107410.

Manandhar, G., P. Sutovsky, H.C. Joshi, T. Stearns, and G. Schatten. 1998. Centrosome reduction during mouse spermiogenesis. *Dev Biol*. 203:424-434.

Santos, P.R., M.F. Oliveira, M.A. Arroyo, A.R. Silva, R.E. Rici, M.A. Miglino, and A.C. Assis Neto. 2014. Ultrastructure of spermatogenesis in Spix's yellow-toothed cavy (Galea spixii). *Reproduction*. 147:13-19.

Soley, J.T. 2016. A comparative overview of the sperm centriolar complex in mammals and birds: Variations on a theme. *Animal reproduction science*. 169:14-23.

Steppan, S.J., and J.J. Schenk. 2017. Muroid rodent phylogenetics: 900-species tree reveals increasing diversification rates. *PLoS One*. 12:e0183070.

Van Der Horst, G., L. Maree, S.H. Kotze, and M.J. O'Riain. 2011. Sperm structure and motility in the eusocial naked mole-rat, Heterocephalus glaber: a case of degenerative orthogenesis in the absence of sperm competition? *BMC Evol Biol*. 11:351.

Woolley, D.M., and D.W. Fawcett. 1973. The degeneration and disappearance of the centrioles during the development of the rat spermatozoon. *Anat Rec*. 177:289-301.

Yanagimachi, R., Y. Kamiguchi, S. Sugawara, and K. Mikamo. 1983. Gametes and fertilization in the Chinese hamster. *Gamete research*. 8:97-117.
