# Supplementary material for: The evolution of centriole degradation in mouse sperm
Source: Nat Commun. 2024 Jan 2;15:117. doi: 10.1038/s41467-023-44411-8 (PMC10761967; doi:10.1038/s41467-023-44411-8)
Supplement: Supplementary file 6 — Reporting Summary [file 41467_2023_44411_MOESM6_ESM.pdf]

Corresponding author(s): *Double-anonymous peer review submissions: write DAPR and your manuscript number here instead of author names.*

Last updated by author(s): *YYYY-MM-DD*

Reporting Summary

Nature Portfolio wishes to improve the reproducibility of the work that we publish. This form provides structure for consistency and transparency in reporting. For further information on Nature Portfolio policies, see our Editorial Policies and the Editorial Policy Checklist.

Statistics

For all statistical analyses, confirm that the following items are present in the figure legend, table legend, main text, or Methods section.

n/a	Confirmed	
☐	☒	The exact sample size (*n*) for each experimental group/condition, given as a discrete number and unit of measurement
☐	☒	A statement on whether measurements were taken from distinct samples or whether the same sample was measured repeatedly
☐	☒	The statistical test(s) used AND whether they are one- or two-sided<br>*Only common tests should be described solely by name; describe more complex techniques in the Methods section.*
☒	☐	A description of all covariates tested
☐	☒	A description of any assumptions or corrections, such as tests of normality and adjustment for multiple comparisons
☐	☒	A full description of the statistical parameters including central tendency (e.g. means) or other basic estimates (e.g. regression coefficient) AND variation (e.g. standard deviation) or associated estimates of uncertainty (e.g. confidence intervals)
☐	☒	For null hypothesis testing, the test statistic (e.g. F, t, r) with confidence intervals, effect sizes, degrees of freedom and P value noted<br>*Give P values as exact values whenever suitable.*
☒	☐	For Bayesian analysis, information on the choice of priors and Markov chain Monte Carlo settings
☒	☐	For hierarchical and complex designs, identification of the appropriate level for tests and full reporting of outcomes
☒	☐	Estimates of effect sizes (e.g. Cohen's *d*, Pearson's *r*), indicating how they were calculated

Our web collection on statistics for biologists contains articles on many of the points above.

Software and code

Policy information about availability of computer code

Data collection	No unreported custom computer code or algorithm was used.<br>We used Leica LAS X program.
Data analysis	No unreported custom computer code or algorithm was used.<br>We used Microsoft word, Microsoft Excel, Adobe photoshop, Adobe illustrator, GraphPad Prism 8.0, PAML suite, HyPhy suite, MUSCLE, AUGUSTUS gene prediction tool, ModelFinder program in IQTree, MrBayes, FigTree, Nikon's NIS-Elements,

For manuscripts utilizing custom algorithms or software that are central to the research but not yet described in published literature, software must be made available to editors and reviewers. We strongly encourage code deposition in a community repository (e.g. GitHub). See the Nature Portfolio guidelines for submitting code & software for further information.

Data

Policy information about availability of data

All manuscripts must include a data availability statement. This statement should provide the following information, where applicable:

- Accession codes, unique identifiers, or web links for publicly available datasets
- A description of any restrictions on data availability
- For clinical datasets or third party data, please ensure that the statement adheres to our policy

Relevant data supporting the findings of this study are available in this manuscript and in the supporting information file. Source data and associated measurements

are provided with this manuscript. Data shown in this manuscript are available from the corresponding author with a reasonable request. We have created a project named "The Evolution of Centriole Degradation in Mouse Sperm" in the Figshar public repository that contains the paper's research data.

Research involving human participants, their data, or biological material

Policy information about studies with human participants or human data. See also policy information about sex, gender (identity/presentation), and sexual orientation and race, ethnicity and racism.

Reporting on sex and gender	The research subject is sperm, which is naturally focused on male fertility.
Reporting on race, ethnicity, or other socially relevant groupings	Unknown
Population characteristics	Unknown
Recruitment	Recruitment was based on availability in UToledo Biorepository Bank.
Ethics oversight	IRB #0000202366

Note that full information on the approval of the study protocol must also be provided in the manuscript.

Field-specific reporting

Please select the one below that is the best fit for your research. If you are not sure, read the appropriate sections before making your selection.

☒ Life sciences ☐ Behavioural & social sciences ☐ Ecological, evolutionary & environmental sciences

For a reference copy of the document with all sections, see nature.com/documents/nr-reporting-summary-flat.pdf

Life sciences study design

All studies must disclose on these points even when the disclosure is negative.

Sample size	The sample size was not predetermined. The sample size is the number of sperm or centrioles analyzed and is described in the figure or figure legend of each experiment.
Data exclusions	No data were excluded.
Replication	Confocal and STORM experiments was repeated at least three times and results were consistent. The final data pool together results from different experiments.
Randomization	We did not randomized the experiments.
Blinding	The researchers were not blinded for this study because of its exploratory nature.

Reporting for specific materials, systems and methods

We require information from authors about some types of materials, experimental systems and methods used in many studies. Here, indicate whether each material, system or method listed is relevant to your study. If you are not sure if a list item applies to your research, read the appropriate section before selecting a response.

Materials & experimental systems

n/a	Involved in the study	
☐	☒	Antibodies
☐	☒	Eukaryotic cell lines
☒	☐	Palaeontology and archaeology
☐	☒	Animals and other organisms
☒	☐	Clinical data
☒	☐	Dual use research of concern
☒	☐	Plants

Methods

n/a	Involved in the study	
☒	☐	ChIP-seq
☒	☐	Flow cytometry
☒	☐	MRI-based neuroimaging

Antibodies

Antibodies used	Antibody, (Production animal) Company (Catalog number), Concentration (Application)<br>POC5 ab1 (Rabbit) Gifted by Dr. Bernan (PMID #19349582) 1:200 (Confocal) 1:100 (STORM)

POC5 ab2 (Rabbit) Thermo Fisher Scientific (PA5-24308) 1:200 (Confocal) 1:100 (STORM)
CETN1 (Mouse) Santa Cruz (2A6) 1:10 (Confocal) 1:5 (STORM)
POC1B ab1 (Rabbit) Thermo Fisher Scientific (PA5-24495) 1:300 (Confocal)
POC1B ab2 (Mouse) Thermo Fisher Scientific (H00282809-B01P) 1:300 (Confocal) 1:100 (STORM)
FAM161A ab1 (Rabbit) Sigma Aldrich (HPA032119) 1:300 (Confocal)
FAM161A ab2 (Rabbit) Novus Biologicals (NBP1-91508) 1:1000 (Western)
FAM161A N-terminus (Rabbit) Gifted by Dr. Dror Sharon (Hadassah-Hebrew University Medical Center) 1:200 (Confocal)
Tubulin (Mouse) DSHB (Developmental Studies Hybridoma Bank) (E7) 1:20 (Supernatant) 1:100 (Concentrate)
Tubulin (Sheep) Cytoskeleton, Inc. (ATN02) 1:500 (Confocal)
Anti-HA (Rabbit) Invitrogen (SG77) 1:200 (Confocal) 1:1000 (Western)
Anti-FLAG (Mouse) Invitrogen (FG4R) 1:400 (Confocal) 1:1000 (Western)
Anti-Rabbit A647 (Donkey) Jackson ImmunoResearch (711-605-152) 1:300 (Confocal) 1:100 (STORM)
Anti-Mouse A647 (Donkey) Jackson ImmunoResearch (715-605-150) 1:300 (Confocal) 1:100 (STORM)
Anti-Rabbit A488 (Donkey) Jackson ImmunoResearch (711-545-152) 1:200 (Confocal)
Anti-Sheep A555 (Donkey) Thermo Fisher Scientific (A-21436) 1:500 (Confocal)
Anti-Mouse A488 (Donkey) Jackson ImmunoResearch (715-545-150) 1:200 (Confocal)
Anti-Sheep A488 (Donkey) Jackson ImmunoResearch (715-545-003) 1:300 (Confocal)

Validation

Describe the validation of each primary antibody for the species and application, noting any validation statements on the manufacturer's website, relevant citations, antibody profiles in online databases, or data provided in the manuscript.

Eukaryotic cell lines

Policy information about cell lines and Sex and Gender in Research

Cell line source(s)	U2OS cells were purchased from American Type Culture Collection
Authentication	American Type Culture Collection
Mycoplasma contamination	No
Commonly misidentified lines (See ICLAC register)	Nothing Found

Animals and other research organisms

Policy information about studies involving animals; ARRIVE guidelines recommended for reporting animal research, and Sex and Gender in Research

Laboratory animals	No live animals were used - Ejaculated spermatozoa were donated by Dr. Bo Harstine at Select Sires Inc. (bovine, Holstein) and Dr. Jie Xu at the University of Michigan (New Zealand rabbit).
Wild animals	Not applicable.
Reporting on sex	The research subject is sperm, which is naturally focused on male fertility.
Field-collected samples	Not applicable.
Ethics oversight	Tissues were collected from discarded euthanized research animals from the University of Toledo, University of Maryland, and the University of Michigan or purchased from a slaughterhouse.

Note that full information on the approval of the study protocol must also be provided in the manuscript.
